# The Heterogeneous Dynamics of Economic Complexity

**DOI:** 10.1371/journal.pone.0117174

**Published:** 2015-02-11

**Authors:** Matthieu Cristelli, Andrea Tacchella, Luciano Pietronero

**Affiliations:** 1 ISC-CNR, Institute for Complex Systems, Rome, Italy; 2 Physics Department, Sapienza University of Rome, Rome, Italy; 3 London Institute for Mathematical Sciences, London, United Kingdom; Feng Chia University, TAIWAN

## Abstract

What will be the growth of the Gross Domestic Product (GDP) or the competitiveness of China, United States, and Vietnam in the next 3, 5 or 10 years? Despite this kind of questions has a large societal impact and an extreme value for economic policy making, providing a scientific basis for economic predictability is still a very challenging problem. Recent results of a new branch—Economic Complexity—have set the basis for a framework to approach such a challenge and to provide new perspectives to cast economic prediction into the conceptual scheme of forecasting the evolution of a dynamical system as in the case of weather dynamics. We argue that a recently introduced non-monetary metrics for country competitiveness (fitness) allows for quantifying the hidden growth potential of countries by the means of the comparison of this measure for intangible assets with monetary figures, such as GDP *per capita*. This comparison defines the fitness-income plane where we observe that country dynamics presents strongly heterogeneous patterns of evolution. The flow in some zones is found to be laminar while in others a chaotic behavior is instead observed. These two regimes correspond to very different predictability features for the evolution of countries: in the former regime, we find strong predictable pattern while the latter scenario exhibits a very low predictability. In such a framework, regressions, the usual tool used in economics, are no more the appropriate strategy to deal with such a heterogeneous scenario and new concepts, borrowed from dynamical systems theory, are mandatory. We therefore propose a data-driven method—*the selective predictability scheme*—in which we adopt a strategy similar to the *methods of analogues*, firstly introduced by Lorenz, to assess future evolution of countries.

## Introduction

Which are the key ingredients determining the economic performance of a country and its future development? Economists traditionally measure the performance with monetary figures such as the gross domestic product (GDP) reflecting, at most, the actual status of a country [1]. The assessment of the development, evolution and growth of countries is instead highly controversial [2–12]. Many intangible elements are therefore invoked such as good education, financial status, labour cost, high tech industry, energy availability, quality of life, etc. However, these concepts are usually discussed in a qualitative way.

A longstanding objective of macro-economic theories is the development of predictive schemes in order to give a quantitative assessment of the future evolution of economic indicators, such as income, Gross Domestic Product (GDP), inflation rate, etc and to provide criteria and indications for economic interventions, stimuli, growth incentives, etc. It may appear surprising that country growth forecast, even if crucial for the wealth of nations and people, is still a controversial open question, as witnessed by the recent critical analysis of Refs. [13, 14].

In this paper we discuss how a non-monetary metrics, able to assess the competitiveness of countries by measuring intangible assets of the economic system strength (fitness) and recently introduced in Refs. [15–17], can provide a fundamental analysis of the hidden potential of growth for countries (see also S1 Information and the section *Materials and Methods* for further discussions on this method). This metrics carries a non-monetary information content on the level of development and competitiveness of economic systems. It captures features of the observed nestedness of the country-product network, which are common in competitive environments, in economics as in biology [18–25]. This approach is based on the idea that the productive basket of a country is able to discount and reflect (almost) all the information encoded in the intangible assets, usually hardly modelable. From a conceptual point of view, it resembles the so-called efficiency property of financial markets where, at least in principle, prices should reflect all the available sources of information.

When we compare monetary figures, as the GDP *per capita*, with this metrics for country intangibles, forecasting economic growth faces issues very similar to those of weather forecasts and, in general, conceptually resembles the challenge of forecasting the evolution of a dynamic system. In this perspective, we are going to show that there exists a strong evidence of a high degree of heterogeneity in the dynamics of countries in the plane defined by the fitness and the GDP *per capita* (hereafter we will use income and GDP *per capita* in an equivalent way). This observation calls for a completely new framework for the predictability criteria making regression-based approaches inappropriate to address the heterogeneous dynamics of the country growth. We propose to call this new approach *selective predictability scheme*. Loosely speaking, this predictive scheme recalls concepts from dynamical systems such as effective dimensions of the phase space and the *methods of analogues* [26, 27] which are typical tools in scenarios in which the laws of evolution are unknown and only a set of empirical observations of the evolution is known. Depending on whether a country has a lower or higher level of income compared to expectations based on its level of fitness, we are able to detect strong and stable evolution trends of countries in specific regimes. We stress the fact that the method we are proposing opens new paths for economic forecasting techniques, concretely data-driven and qualitatively different from standard methods. In particular, the method here proposed follows a strategy radically different from usual large dimensional factor models such as diffusion index forecasting [28] and augmented factor models. As mentioned, instead of a direct search for drivers and determinants of the growth or the construction of effective indices (i.e principal values analysis), we define a synthetic measure on the economic dimension which, in our modeling, discounts the information encoded in the network of capabilities and in their mutual interaction: the productive capacity. Products (proxied by exports) *discount*, in a loose sense of the word, the information on all the determinants of the growth and therefore are the proper level to extract this information content on the whole. From a conceptual point of view, production acts somehow similarly to financial prices which *should* discount all the available information of markets and therefore as prices, in our framework, products are a special set of variables which comprehensively reflect the intangibles we want to measure.

The emerging picture from the *selective predictability scheme* also suggests that, rather than simply overcoming money-based measure of wealth and development (namely GDP) by substituting them with new indicators [29, 30], a more scientific approach would consist in complementing GDP-based measures with new dimensions. In such a way it would be possible to compare purely monetary information with non money-driven indicators to detect informative content about intangible features. Mathematically speaking, this corresponds to project the economic dynamics of country evolution onto a suitable multi-dimensional problem, as done in the case of the fitness-income plane, rather than simply projecting on a one-dimensional indicator alternative to the GDP. It is also worth noticing that such substitutive approaches usually face the issue of mixing heterogeneous indicators whose commensurability is often questionable and problematic.

**Fig 1:**
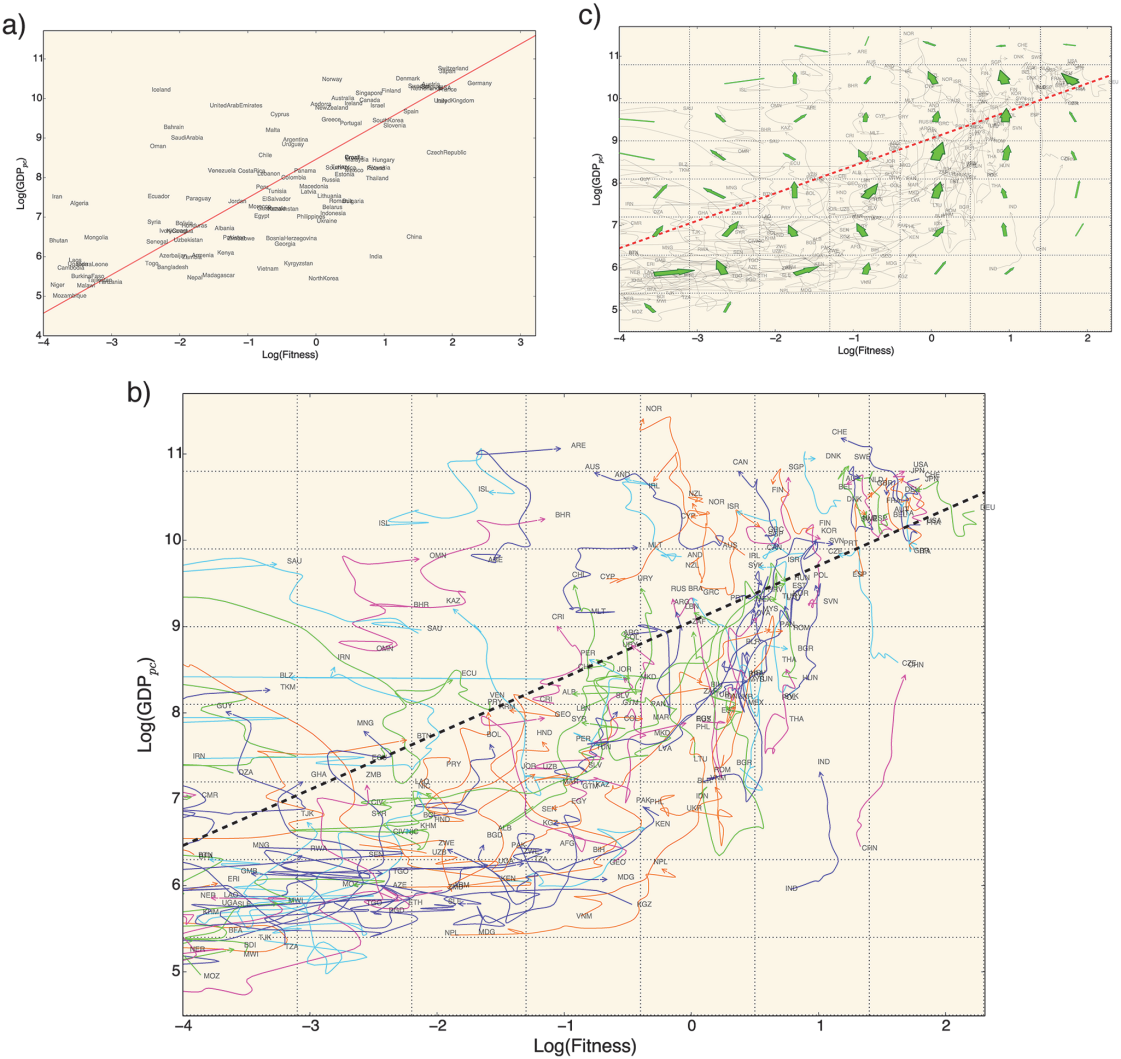
Non-stationary sdynamics of economic systems in the fitness-income plane. a: we report in the fitness-income plane the position of the countries in 1995. The red line indicates the expected level of income, given the level of fitness of a country, and it is the result of the minimization of the Euclidean distance of the countries from the line weighted by the country GDP. b: evolution in the fitness-income plane from 1995 to 2010. We observe a strongly heterogeneous dynamics of the countries in this plane. In order to point out emergent trends in this dynamics, we perform a coarse graining of the trajectories, as shown in panel c. A laminar-like regime is observed. With respect to the evolution of the countries with intermediate/large fitness, this regime is characterized by a regular flow and an income lower than what expected from the average red line (top left corner).

The features of the economic evolution of countries in the fitness-income plane are the starting point of our analysis. As shown in Fig. 1 and S1 Information, a first valuable result is that countries in this plane are not evolving to an equilibrium situation—the equilibrium would correspond to the case in which all countries are on a straight line in the fitness-income plane—at least on the time horizon investigated, i.e. 1995–2010. As a consequence of this fact, the first observation is that the distance between the real income of countries and the expected one is not directly a measure for the potential of growth of countries. This means that both the fitness and this distance are not good candidate as regressors in a standard approach. For further considerations about the poor performances of a regressive approach with these variables, see S1 Information. However, as argued, the features of the evolution of countries in the fitness-income plane call for a scheme in which regressions are no longer the appropriate tool to address the issue of GDP growth forecast.

Standard approaches traditionally assume the existence of overall trends to uncover. In details, the assumption underlying a regression-based approach as the correct analysis tool, is that the system responds homogeneously to a specific set of (independent) variables, explaining a certain amount of the variance of the dependent variable. In other words, regressions may be appropriate tools to analyze systems whose dynamics is homogeneous.

To investigate how income depends on fitness and vice versa, we must move from a static picture to a dynamical investigation of the countries in the income-fitness plane. In Fig. 1b we represent the income-fitness plane with the trajectories of all countries from 1995 to 2010. A better understanding of the dynamics in this plane can be achieved by a coarse-graining of the trajectories. We build a vector-like representation (like a weather forecast map or a fluid vector field) of the movements in the income-fitness plane by dividing the plane in a grid, as shown in Fig. 1c. Each arrow represents the average of all 1-year displacements whose starting point belongs to the box of the grid corresponding to the arrow.

**Fig 2:**
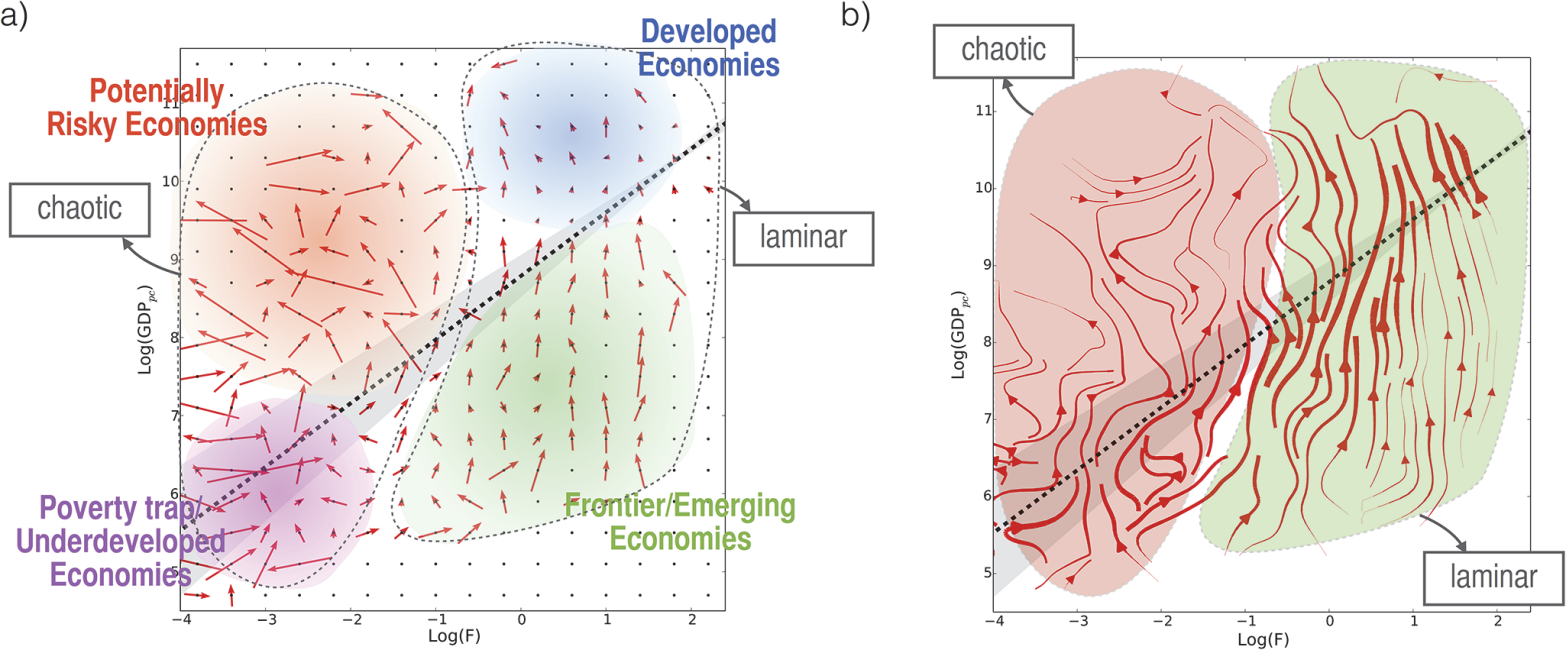
The four regimes of the heterogeneous dynamics of economic complexity. a) A finer coarse graining of the dynamics highlights two regimes for the dynamics of the evolution of countries in the fitness-income plane. There exists a laminar region in which fitness is the driving force of the growth and the only relevant economic variable in order to characterize the dynamics of countries. We argue that the evolution of countries in this region is highly predictable. There is also a second regime, which appears to be chaotic and characterized by a low level of predictability. In the laminar regime we also find two different kinds of evolution patterns for the emergent countries and developed ones respectively. In this heterogenous scenario for the economic dynamics of countries, regressions are no more the appropriate tool to develop a predictive scheme, which instead must face issues which are very close to the problems of predictability for dynamical systems (i.e. atmosphere, climate, wind, ocean dynamics, and weather forecast, etc). b) we report a continuous interpolation of the coarse grained dynamics to better illustrate the two regimes of predictability,

The analysis of country evolutions in the income-fitness plane reveals a strongly heterogeneous behavior (Fig. 1b-c, and Fig. 2). We observe the emergence of different regimes of country evolution and development, depending on the relative value of fitness and wealth. On this account, according to the position in the income-fitness plane, we are able to distinguish two main regimes (see Fig. 2): a laminar-like regime, in which the dynamics appears to be predictable and informative of the country growth (green and blue shades) and a chaotic-like regime in which no clear emergent pattern is observable (purple and red shades). A more careful analysis reveals an even richer ecology and at least four different types of dynamics can be uncovered:

- **Very low fitness regime (Fig. 2 purple area)**: countries are stuck into a poverty trap. Their industrial competitiveness is irrelevant with respect to many other exogenous factors.
- **Low/intermediate fitness regime (Fig. 2 red area)**: for these countries industrial competitiveness is still scarcely relevant with respect to other exogenous factors, which, in this case, have a positive effect. Most of the exporters of heavy natural resources lie in this area.
- **Intermediate fitness regime and income lower than expected (Fig. 2 green area)**: it appears that the fitness is the main driving force for economic growth.
- **High fitness and high income regime (Fig. 2 blue area)**: this region corresponds to developed countries, the flow is still laminar in the income-fitness plane but the dynamics, even if predictable, is of a different kind with respect to the previous regime.

The heterogeneity of the income dynamics in relation to the value of the fitness has several conceptual and practical consequences on how to carry out a predictive scheme for the trajectory evolution. As mentioned, regressions are appropriate tools when we observe homogeneous responses to specific variables but they are not effective in such a heterogeneous scenario. On one hand, in the chaotic-like region of the income-fitness plane there is no clear dynamic relationship between the two variables. On the other hand, even in the laminar-like regime—the intermediate fitness regime with income lower than expected and the high fitness regime—we observe two different types of behavior across emerging and developed countries.

We therefore propose to define a *selective predictability scheme* in which the degree of predictability of the economic dynamics depends on the specific position in the income-fitness plane. The finding of Fig. 2 leads to the observation that this scheme faces issues which are very close to the problem of forecasting the evolution of dynamical systems (i.e. atmosphere, climate, wind, ocean dynamics, and weather forecast, etc) when the laws of motion are unknown. We believe that the present framework opens new paths towards providing more scientific basis for economic predictability.

## Results

### How to predict the heterogeneous dynamics of the economic complexity: the *Selective Predictability Scheme*

**Fig 3:**
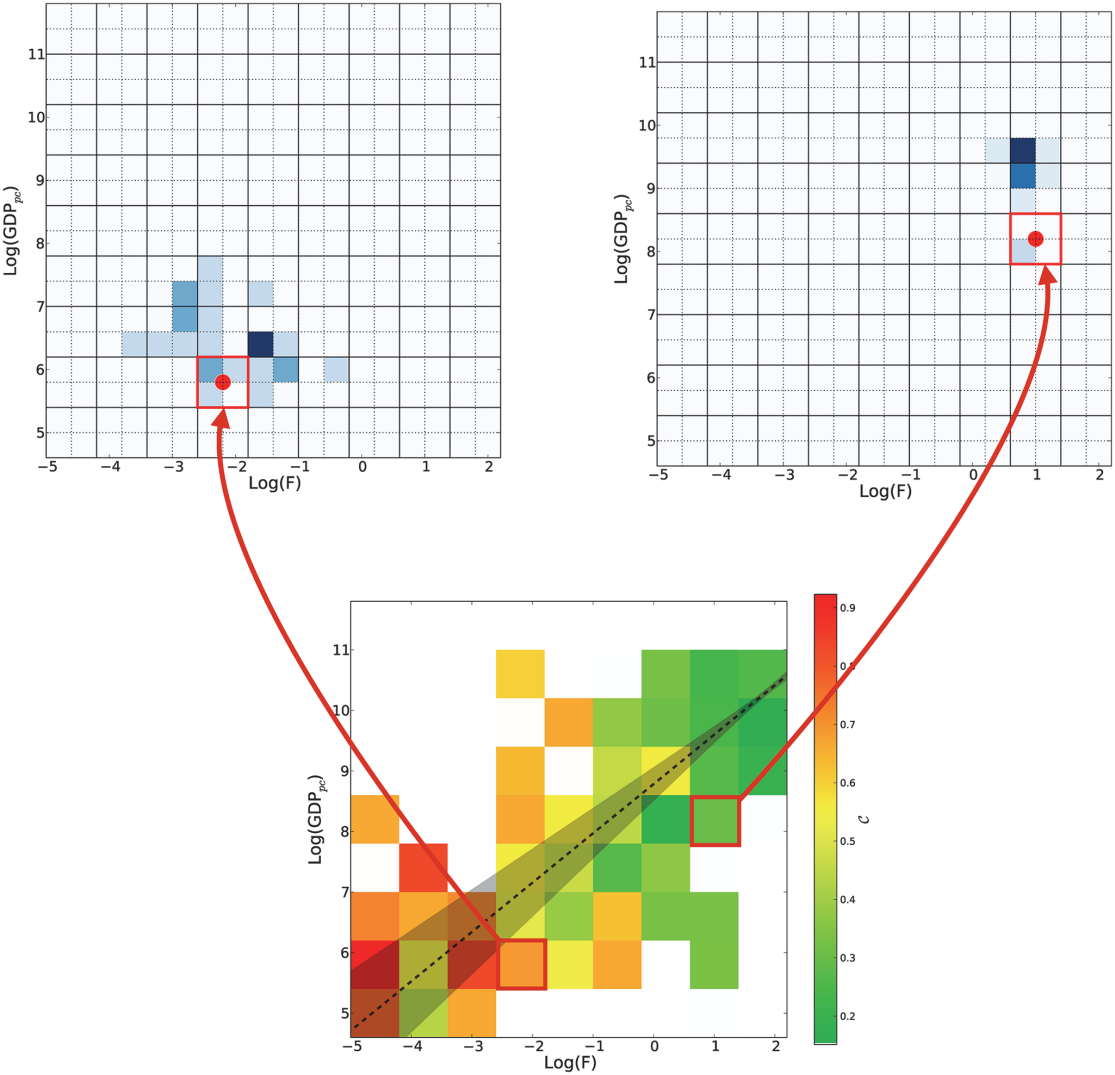
The *selective predictability scheme*. The scheme is based on the *method of analogues* [26, 27] which is a strategy to predict the future from the knowledge of the past. We track the evolution of the countries in a given box and we record where these countries have evolved in the following 10 years. By carrying out systematically this procedure for each box, we build the empirical distribution of the 10-years evolution of countries. We report the empirical 10-years distributions (ED) for two boxes: the former from the chaotic-like regime and the latter from the laminar-like one, as defined in Fig. 2. A visual inspection reveals that the EDs from the chaotic-like regime tend to have a larger dispersion than the ones from the laminar one, which are instead very concentrated in few boxes. To quantify this effect we introduce a measure of concentration for the EDs: $C=(n_{boxes}^{\left(i\right)}/N^{\left(i\right)}-1/N^{\left(i\right)}){\left(1-1/N^{\left(i\right)}\right)}^{-1}$, where $n_{boxes}^{\left(i\right)}$ and *N* ^(*i*)^ are respectively the number of occupied boxes by the ED associated to the box *i* and the number of points giving rise to ED. This measure, see S1 Information for further considerations, confirms the existence of two regimes characterized by two very different levels of predictability: a laminar regime (green boxes) for which the flow is regular and tends to be concentrated in few boxes and a chaotic regime characterized by very dispersed distributions of the country evolution. We argue that in the first regime the fitness is the key ingredient to develop a forecasting scheme for the evolution of the economic systems.

Let us now discuss the results of Fig. 2, borrowing concepts and methods from the theory of dynamical systems. In terms of the jargons of dynamical systems [26, 27, 31], in the laminar regimes (green and blue shades of Fig. 3), we argue that the effective dimension of the phase space of the dynamics is approximately two and, in this perspective, we are looking at the right two dimensions for the dynamics. In other words, the economic dynamics is, in general, a highly dimensional problem but in the laminar regime the effective dimension of the phase space of this dynamics is much lower. Differently for the chaotic-like regime we can find two different explanations.

On one hand, it could be that the effective dimension of the phase space is still two and the dynamics is indeed chaotic.

On the other hand, it might be that in this region the dimension of the phase space of the dynamics is much larger than two. Therefore the fitness-income plane is the projection of the *d*-dimensional dynamics onto a two-dimensional space. The dynamics appears to be chaotic-like, as the result of the large dimension of this space, because we are not able to see any real recurrences, as intended by Poincaré in his theory [32]. According to this second interpretation, trajectories which appear close in the projected two-dimensional space are instead, differently from points and trajectories in the laminar-like regime, very far in the real *d*-dimensional (d ≫ 2) space. Consequently these trajectories are only apparently good candidate to be *analogues*, that is close points in the whole space of the evolution. Roughly speaking, the higher is the dimensionality of the space phase, the harder is to find a good analogue—a point close enough in the phase space—to infer the future from the past.

Translating these arguments in economic terms, we believe that the latter interpretation better fits our scenario. In the laminar-like regime, the fitness is the relevant economic variable in order to understand the dynamics of the income and, in general, of the growth of the GDP. In the other regime, instead, the dynamics is ruled by several exogenous factors which compete with the fitness in driving the evolution of countries. Close points in the chaotic regime are likely ruled by very different economic regimes given their real distance in the complete space of the economic dynamics.

It results that the predictive scheme, required by the dynamics of economic complexity, is analogous to the problem of predicting the future of a dynamical system in the case in which we do not know the equations of motion (i.e. the rule of the evolution). The best strategy is therefore to try to predict the future from the knowledge of the past: this method is called *method of analogues* and was firstly introduced by Lorenz [26, 27] (see also the S1 Information).

We want to stress that the systematic and formal investigation of the causality dependence is beyond the scope of the present work in which, instead, we are interested in developing a scheme for the growth forecast. Anyway, it is clear that this causality analysis represents the natural evolution of the present research and a recently introduced method, called *convergent crossing maps* [33] appears to be an ideal candidate to carry out such task. It is worth noticing that, once again, this tool arises from dynamical systems discipline and extends the realm of causality estimation to a wider class of systems with respect to standard causality test (i.e. Granger causality).

As a first step to implement a predictive scheme inspired by the method of analogues, we investigate the 10-years growth of the GDP *per capita* in the income-fitness plane (in the S1 Information we also discuss the 5-years growth case for which similar results hold).

Following the line of reasoning of Fig. 2, we can perform a coarse graining of the trajectories dividing the plane into square boxes. Let us now suppose that in a specific year we find a given number of countries in a given box and we record where these countries evolve in the following *n*-years—as mentioned, we now discuss the case for *n* = 10 years. A detailed discussion on methods, calculations, definitions and robustness of the findings about the *selective predictability scheme* are reported in the section *Materials and Methods* and in the S1 Information. By carrying out systematically this procedure for each box, we can build the empirical distribution of the 10-years evolution of the countries, box-by-box. We report the empirical 10-years distributions (ED hereafter) for two boxes from the chaotic-like regime and the laminar-like regime respectively in Fig. 3 (top panels) (a larger set of EDs are reported in the S1 Information). Confirming the heuristic observations drawn in Fig. 2, we observe that the dispersion of the EDs tends to be larger in the chaotic-like regime than in the laminar-like one. Let us try to quantify the degree of predictability of the 10-years evolution in this plane for each box by measuring the concentration of the EDs we have obtained. The simplest way to define such a concentration is by counting the number of occupied boxes, using the ED associated to a given box *i* and normalizing the results with the total number of points in the box *i*. We would therefore obtain the highest concentration, and therefore the highest predictability from our point of view, if all the countries in a box would evolve in the same arrival box. As for information theory entropy which is zero when the system is completely predictable, we then define our concentration measure as $C=(n_{boxes}^{\left(i\right)}/N^{\left(i\right)}-1/N^{\left(i\right)}){\left(1-1/N^{\left(i\right)}\right)}^{-1}$, where $n_{boxes}^{\left(i\right)}$ and *N* ^(*i*)^ are respectively the number of occupied boxes by the ED associated to the box *i* and the number of points giving rise to ED. In Fig. 3 bottom panel, we show the predictability for each box as measured by 1-*C* (see section Materials and Methods and S1 Information for further considerations on this point). In general, we find that all the concentration/predictability measures we have tested confirm the heuristic visual grouping discussed in Fig. 2: there exists a region of the fitness-income plane in which the dynamics is laminar and is characterized by a high degree of predictability, by contrast the chaotic region exhibits broader EDs with a large dispersion corresponding to a lower degree of predictability of the dynamics.

At this stage, considering the 1995–2010 evolution, we have confirmed that there exist regions for which the EDs are very concentrated. Now we want to test if the EDs built from past evolution of countries are a good tool to assess the issue of the growth forecast. Back into the jargons of dynamical systems, if we have a box in a region for which the effective dimension of the economic dynamics is close to 2—fitness is the only driving variable for the dynamics of country development—we expect that the ED of this box is a good proxy for evaluating the evolution of a country. In fact in such a case, the ED would be estimated from a set of points which are good candidates to be analogues. These points are not only close in the fitness-income plane but also in the real space of the economic dynamics and therefore they are ruled by a similar economic regime, even in the projected space. It is worth noticing that we are also assuming that the EDs vary on a time horizon longer than the one under investigation. We expect that the boxes with highest predictability, as measured by the concentration *C* in Fig. 3, are also the regions for which we expect EDs to be reliable instruments to predict growth of countries. Such a framework does not merely forecast the GDP growth (with an appropriate uncertainty) but even the future evolution of the country’s trajectory in the fitness-income plane.

**Fig 4:**
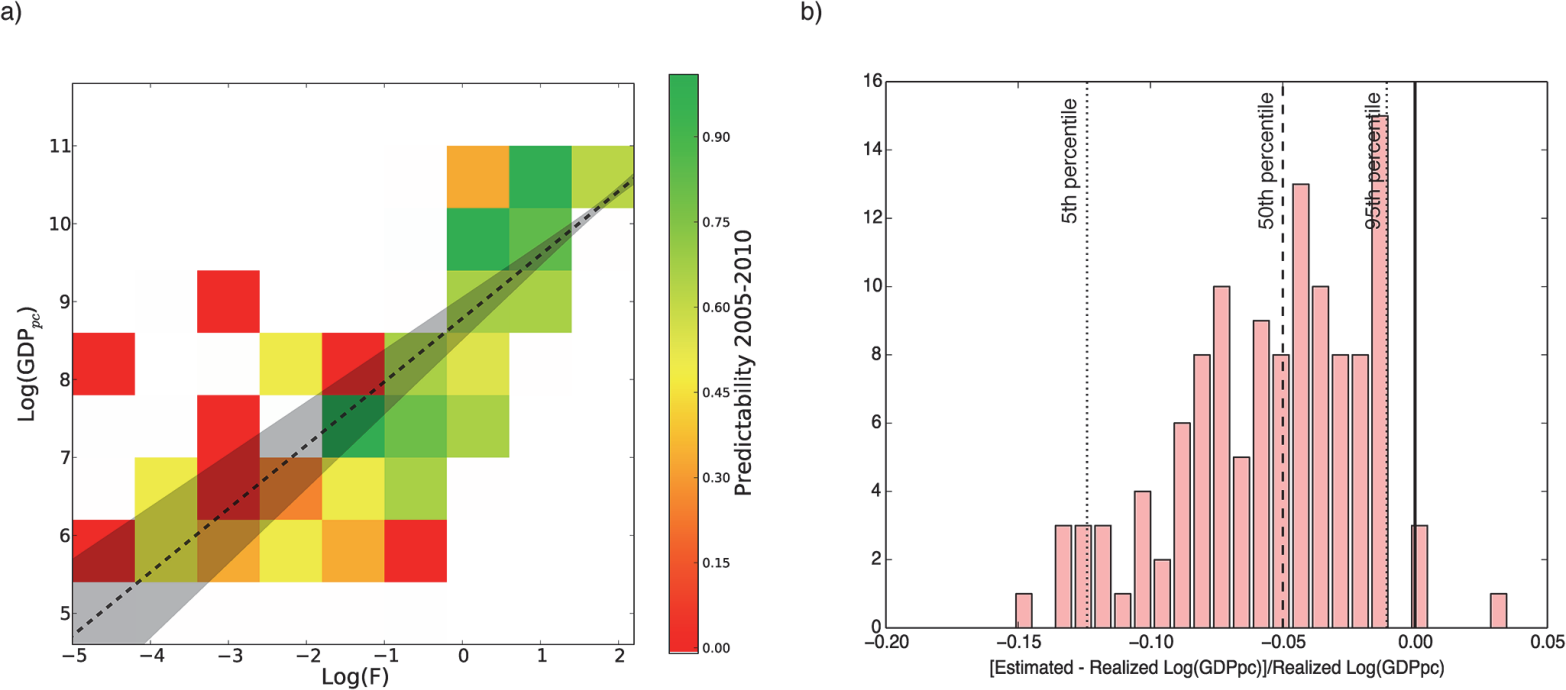
Back testing of the 5-years *Selective Predictability* Scheme. a), we train the EDs on the time interval 1995–2005. We test the rate of success of the forecast of the position of a country in 2010, according to the ED associated to the departure box of the country in 2005. We compute how many times we are able to guess correctly the box in which the country will be after 5 years. Although the very limited statistics of this back testing procedure, the results confirm the fact that, not only the forecasted area in which we expect to find the country is smaller in the laminar regime, as demonstrated by Fig. 3, but even the dynamics appears to be more predictable because of a general higher rate of success of the *selective predictability scheme* in the laminar region. b), we report the histogram for the relative error of the forecast of 2010’s GDP *per capita*. Differently from the fitness histogram (not reported), the distribution is not peaked around 0. We believe this systematic under evaluation of growth is due to a training set shorter than the typical length of an economic cycle.

To verify this hypothesis, we must perform a back-test analysis of our methodology. Given the time length of our dataset, we cannot perform the training of our predictive scheme on a 10-years horizon because we would not have enough years in order to consider an independent out-of-the-sample set of 10 years. We therefore back-test our method on the 5-years time horizon and we use our series in the following way: we train the method on the period from 1995 to 2005 and we build the 5-years EDs for each box. Once the 5-years EDs are estimated, we test how successful they are in forecasting the evolution of countries in the period 2005–2010. The results, shown in Fig. 4 (panel a), confirm our arguments and the existence of high predictable regimes, where our scheme is effective and of low predictable regimes, where the fitness is no more the key driving ingredient of the development of countries. This overall picture for the economic dynamics and its heterogeneity could not be properly grasped by a regression-based approach. In panel b) of Fig. 4, we report the relative error of 2010’s GDP *per capita* forecast. We observe a systematic underestimation of the growth. We argue that the reasons are twofold: on one hand, the training period is shorter than a complete economic cycle and therefore we are estimating future growth with an incomplete set of information. On the other hand, we expect the systematic over or under estimation to be less likely observed on longer time-horizon. The same histogram for fitness (not reported) instead tends to be substantially bell-shaped and peaked around 0. See section Materials and Methods and S1 Information for further details on the method used to estimate the 2010’s GDP *per capita*.

We can also conceive a more refined setup to implement the selective predictability scheme. In particular, up to now, we have used a sort of *Eulerian* specification of the flow of the country evolution [34]. As discussed, this approach permits to give both a global representation of the features of the economic regime of country evolution and a tool to forecast country evolution. However, when a finer country-specific projection is needed, we can think of a sort of Lagrangian specification of the country evolution [34]—the scheme here proposed but applied to a neighborhood of a country in the fitness-income plane and not to a box independently on the country position—though this represents a technical rather than a conceptual refinement.

## Discussion

Are development, wealth and growth only a matter of GDP? In the last decade, a growing literature [30] is trying to overcome a description of economic systems in purely GDP-oriented terms, by substituting GDP with new economic indicators. However, here we argue that economic systems are unavoidably *also* a matter of monetary information, given the organization and the rules of modern economies. It would be therefore *naif* to simply neglect monetary dimension in the attempt of assessing the development and wealth of countries. We believe a more scientific grounding of a new economic thinking would consist in a line of reasoning close to the one proposed in this paper: comparing monetary information with measures of intangible assets of countries. In this paper we have shown that the growth dynamics of countries in the fitness-income plane exhibits a high degree of heterogeneity and that regression-based analysis are consequently no longer the appropriate tools for developing a predictive scheme. We argue instead that techniques and methods deriving from the dynamical system theory appear as natural candidates to explain and model the complex dynamics in this plane. One of the main consequences of this approach is that we observe a heterogeneous degree of predictability, depending on the region of the plane considered.

The scheme that we propose for the prediction of the heterogeneous dynamics of the economic complexity resembles the so-called *method of analogues*, which is a method developed to predict the evolution of a system (typically a dynamical system) given the observation of the past and without the knowledge of the equation of the dynamics. This conceptual framework is also able to give some insights at the base of a regime-dependent predictability of economic evolution. We know from dynamical systems theory that the limit of application of this method relies on the dimension of the phase space of the dynamics. We argue that only in the laminar-like region such analysis can be effective since the effective dimension of the space is approximately 2. In this regime, economically speaking, the fitness is the driving and dominant variable for understanding the growth of countries.

As a final point, we stress the generality of the proposed approach, which can be extended to the analysis of the dynamics of many economic and demographic indicators. This work points towards the development of forecasting methodologies and techniques which have a stronger scientific grounding than standard approaches used in economics.

Last but not least, we also observe that the formulation in terms of dynamical systems opens a clear strategy to solve the problem of estimating causality relations from the the observation of simple correlations in a scenario where, differently from physics and other natural sciences, it is hard to pinpoint cause-effect relations among variables. The investigation of the causality relation between fitness and growth represents the natural next step of this research. On this account the method introduced in [33] appears as the suitable tools to uncover the non-trivial relationship between the ecosystem of capabilities and the growth regime.

## Materials and Methods

In this section, we provide the description of datasets and methodologies used in this work. Additional materials and discussions are also reported in the S1 Information where we also address the robustness of the methodology and of our findings.

### Datasets and definition of the binary country-product matrix

Concerning export data, we use data extracted from the BACI dataset [35]. This dataset is, in turn, grounded on the COMTRADE dataset (freely accessible from the UN Comtrade website [36]) and is the result of a reconciliation procedure extensively discussed in [35]. In this dataset, we have trading data about more than 200 countries and 5000 products classified according to a six digit code (categorization: Harmonized System 2007 [37]). Data spans the time interval from 1995 to 2010 on a yearly basis. We have coarse-grained such classification by considering only the first 4 digits, obtaining a set of 1131 products. The matrix *M*, whose elements are *M*_*cp*_, is then built by transforming the flows *q*_*cp*_ of US Dollars into unweighted links between countries and products. The criterion adopted in order to determine whether a country can be considered a producer or not of a particular product is the so-called Revealed Comparative Advantage (RCA) [38] that is the fraction of product *p* in the export basket of country *c* with respect to the fraction of the total export of product *p* in total world export. This quantity is then divided by the fraction of the total export of *c* with respect to the whole world export, i.e.:

$$RCA_{cp}=\frac{\frac{q_{cp}}{\sum_{p^{\prime }}\;q_{cp^{\prime }}}}{\frac{\sum_{c^{\prime }}\;q_{c^{\prime }p}}{\sum_{c^{\prime }p^{\prime }}\;q_{c^{\prime }p^{\prime }}}}.$$

In order to build the binary matrix *M* from the *RCA* matrix, we consider *M*_*cp*_ = 1 if *RCA*_*cp*_ ≥ 1 and zero otherwise.

GDP, GDP *per capita* and population are instead extracted from the World Bank dataset [39].

### Definition of country fitness and product complexity: motivation and methodology

The motivation underlying the non-monetary metrics for country competitiveness derives from the recent observation that mainstream theories about economic growth appear not to properly describe the origin of the wealth and competitiveness of countries [15, 16, 40, 41]. The analysis of the export basket of countries [15, 16, 40] reveals that the wealthiest and most competitive economies are usually those which exhibit the highest diversity of export. On the other hand, poorly diversified economies tend to be specialized on those few products which are exported by almost all other countries. Therefore, export data reveals a structure usually defined as nested.

Clearly, specialization and diversification are both involved in the development of the country wealth and, *a priori*, they are both possible candidates to be the key drivers of growth and development. However, empirical observations support the idea that, in a globalized and dynamic scenario, diversification turns to be a far more important element than specialization in the determination of the fitness of an economic system [15].

In such a new perspective, it is therefore crucial to turn into *quantitative* terms the competitive advantage deriving from the diversification. In Refs. [15, 16] a new statistical approach, based on coupled non-linear maps, is developed and its fixed point defines a new metrics for the competitiveness of countries (fitness) and the complexity of products. This novel non-monetary metrics, differently from previous attempts [40], corresponds, given the paradigm of economic complexity, to the simplest and coherent approach to assess the competitiveness of economic systems. The key point to properly address this issue is the non-linear coupling which must hold between country fitness and product complexity. This non-linear coupling translates into mathematical terms the nested structure of the country-product bipartite network. In detail, on one hand, a diversified exporter gives very limited information on the complexity of the product itself, because this kind of countries exports almost all products. On the other hand, when a poorly diversified country is able to export a specific product, very likely this product requires a low level of sophistication. Therefore a non-linear and extremal relationship is required in order to bound the complexity of products by the fitness of the less competitive countries exporting them. For further details on the mathematical details of the definition of the metrics see Refs. [15, 16].

In order to implement this methodology, we define an iterative process which couples the fitness of a country *F*_*c*_ to the complexity of a product *Q*_*p*_. At each step of this process, the fitness *F*_*c*_ is proportional to the sum of the products exported—diversification—weighted by their complexity *Q*_*p*_. Concerning the product complexity *Q*_*p*_, the situation is more subtle. To a first approximation, the complexity of a product is inversely proportional to the number of countries which export it. In addition, if a country has a high fitness, this should reduce the weight in bounding the complexity of a product, and the countries with low fitness should strongly contribute to the bound on *Q*_*p*_. The simplest way to summarize these ideas in mathematical terms is the following iterative scheme:

$$\{\begin{array}{ccc} {\tilde{F}}_{c}^{\left(n\right)} & = & \sum_{p}M_{cp}Q_{p}^{\left(n-1\right)}\\ {\tilde{Q}}_{p}^{\left(n\right)} & = & \frac{1}{\sum_{c}M_{cp}\frac{1}{F_{c}^{\left(n-1\right)}}} \end{array}\to \{\begin{array}{ccc} F_{c}^{\left(n\right)}=\frac{{\tilde{F}}_{c}^{\left(n\right)}}{{\langle {\tilde{F}}_{c}^{\left(n\right)}\rangle }_{c}} & & \\ Q_{p}^{\left(n\right)}=\frac{{\tilde{Q}}_{p}^{\left(n\right)}}{{\langle {\tilde{Q}}_{p}^{\left(n\right)}\rangle }_{p}}. & & \end{array}$$

This iterative method is composed of two steps at each iteration: we first compute the intermediate variables ${\tilde{F}}_{c}^{\left(n\right)}$ and ${\tilde{Q}}_{p}^{\left(n\right)}$ and then we normalize them. The elements *M*_*cp*_ are the elements of the binary country-product matrix *M* previously discussed. We define the metrics for the country fitness and the product complexity as the fixed point of this process.

The stability and the robustness of these metrics are extensively discussed in [16] and [42] respectively. On one hand, it is possible to show numerically that the fixed point of these coupled maps is unique, stable and not dependent on the initial condition, at least when the initial condition belongs to the set of conditions which are meaningful from an economic point of view. This corresponds to test the stability of the method only for those initial conditions satisfying $\sum_{c}F_{c}^{\left(0\right)}=C$, $\sum_{p}Q_{p}^{\left(0\right)}=C$ and all *Q*_*p*_ and *F*_*c*_ cannot be zero, where *C* is a constant and, without loss of generality, *C* = 1. In this way, the normalization we enforce at each step of the process is also satisfied by the initial condition. The only feature dependent on this set of initial conditions is the convergence time towards the asymptotic solution. On the other hand, the methodology appears to be very stable even for noisy dataset as discussed in Ref. [42] given its non-linear structure.

### Coarse-graining of the country evolution in the fitness-income plane

In order to perform the coarse-graining of the country evolution from 1995 to 2010 shown in Fig. 2, we first build a grid in the fitness-income plane. For this figure, we used square boxes with side length equal to 0.4. We observe that the heterogeneous dynamics emerging from this procedure is robust varying the shape and the size of the box in a large rage of values. For each box, we consider all the countries that in a given year are in that box and we compute and plot the mean 1-year displacement for these countries. This means that a country can contribute to the 1-year mean displacement of different boxes. The number of events contributing to different boxes can be very heterogeneous and we consider in our analysis only the boxes with at least 5 events. The robustness of the coarse-grained dynamics is discussed in the S1 Information for different time windows.

### The Selective Predictability Scheme

Similarly to the coarse-graining procedure, in order to train the SPS, we define a grid in the fitness-income plane with square box and side length equal to 0.4. The results of the SPS are robust if the size and the shape of the box are changed. For each box, we consider all the countries that in a given year are in that box and we record the starting position and arrival position after Δ years for these countries, where Δ is the time horizon of the SPS. In the present work, we present the results for Δ = 5 and 10. The evolution distributions (ED), associated to each box, are evaluated on a finer grid and we use a box size which is half the one of the grid defining the box. As a final step, the mean Δ-displacement of each box is defined as the displacement from the center of mass of the starting positions to the center of mass of the arrival points. It is obvious that we can only train the SPS on a time window spanning from 1995 to 2010 - Δ. Finally the forecasted evolutions of the SPS consist in evaluating the box a country belongs to and summing the Δ-displacement to its position in the income-fitness plane.

Further information on the robustness and evaluation of the SPS are provided in the S1 Information.

### Measures of concentration

In order to assess how concentrated are the EDs, we define an *average* measure of concentration, which does not rely on the estimation of the empirical frequencies of the ED as follows

$$C=\frac{n_{boxes}^{\left(i\right)}/N^{\left(i\right)}-1/N^{\left(i\right)}}{1-1/N^{\left(i\right)}}$$

where *N* ^(*i*)^ and $n_{boxes}^{\left(i\right)}$ are respectively the number of events giving rise to the *i-th* ED and the number of boxes in which these *N* ^(*i*)^ evolved after a given time lag. The *C* is a normalized concentration measure since it ranges from 0 to 1. This measure of concentration permits to clearly identify the emergence of two regimes in the fitness-income plane as shown in Fig. 4 and in the S1 Information. We refer to S1 Information for further discussions and motivations supporting the concentration measures used and for comparison with other standard measures such as the entropy and the Herfindahl Index.

## Supporting Information

S1 Information We report and discuss a number of auxiliary results supporting the main findings of the principal paper and assess the robustness of the *selective predictability scheme*. We also provide a discussion of the results which would be obtained by means of standard regressive approaches. In the final part, we also expand the discussion of the validation of the forecasting power of the method through the backtesting procedure proposed.
(PDF)
