# Supplementary material for: The Heterogeneous Dynamics of Economic Complexity
Source: PLoS One. 2015 Feb 11;10(2):e0117174. doi: 10.1371/journal.pone.0117174 (PMC4325004; doi:10.1371/journal.pone.0117174)
Supplement: S1 Information — We also provide a discussion of the results which would be obtained by means of standard regressive approaches. In the final part, we also expand the discussion of the validation of the forecasting power of the method through the backtesting procedure proposed. (PDF) [file pone.0117174.s001.pdf]

Supporting Information S1: The heterogeneous dynamics of economic complexity

Matthieu Cristelli[1,2,*], Andrea Tacchella[2,1], L. Pietronero[1,2,3]
1 ISC-CNR, Institute for Complex Systems, Rome, Italy
2 Physics Department, Sapienza University of Rome, Rome, Italy
3 London Institute for Mathematical Sciences, London, United Kingdom
∗ E-mail: Corresponding matthieu.cristelli@roma1.infn.it

Here we discuss a number of auxiliary results supporting the main findings of the principal paper and assess the robustness of the *selective predictability scheme* (SPS hereafter). We also provide a discussion of the results which would be obtained by means of standard regressive approaches. In the final part, we also expand the discussion of the validation of the forecasting power of the method through the backtesting procedure proposed.

1 Fitness-Income cloud from 1995 to 2010

The deviation of the metrics from the monetary information is the key point to uncover the hidden potential of the growth of countries. The natural candidate for this study is the fitness-income scatter plot (i.e. GDP per capita-fitness plane), as shown in Figs. S1 and S2 where we report the static plot for all the available 16 years of our dataset from 1995 to 2010. The red line represents an estimation of the expected income of a country given its level of complexity. This line is not a regression in the form $GDP = \alpha F + \beta$ but it is the result of the minimization of the Euclidean distance from the line weighted by the country GDP. At this stage, this line does not represent a statement of cause-effect relationship between fitness and income. However, we will show that by looking at the dynamics in this plane we are able to develop a predictive scheme in some specific regimes.

As also shown by the evolution of the residuals of the minimization in Fig. S3, we do not observe any convergence to an equilibrium situation, that is the convergence of the cloud to a straight line in the fitness-income plane. On the opposite, the variance of the cloud tends to increase in the period under investigation. One of the reason of such increase can be traced back in the fact that in the last years of our dataset we observe that China and other emerging countries are eroding the fitness of western countries and overcoming almost all of them. We are somehow in a sort of changing of the drivers or barycenter of the world economy, which is shifting from western developed countries to Asia. In forthcoming works, we plan to deepen the analysis of the overall dynamics of the cloud on a longer time window.

It is worth noticing that the increase of the residuals is not merely due to the increase of the number of countries considered (from 146 to 148) in the time window investigated; we observe, in fact, the same increasing patterns for the residuals even considering constant the number of countries (146). We also want to stress that, in such a framework, we do not expect that the fitness asymptotically will converge to the value of the GDP *per capita* as in an equilibrium scenario. Furthermore, if a static picture and a convergence of the fitness towards GDP *per capita* are assumed, a non trivial issue of the type *why now?* arises, i.e. why in the last 20-30 years the world went out of equilibrium?

2 Fitness *vs* population.

In this section, we briefly discuss how and whether the fitness correlates with the population of a country. In Refs. [1, 2], we have already noticed that the *correct* counterpart of the fitness appears to be a monetary intensive measure, such as the GDP *per capita*, thanks to the observation of the scaling properties of the distribution of the fitness. Here, we confirm and support this observation and interpret the non-trivial residual dependence of fitness on population.

We find that population of a country accounts for a small fraction of the variance (around $3-7\%$ in the range of years here investigated) of the fitness. In Fig. S4 we show the scatter plot in 2004 (the shape appears very similar across years), in that year, the logarithm of the population explains approximately the 6% of the variance of the Fitness.

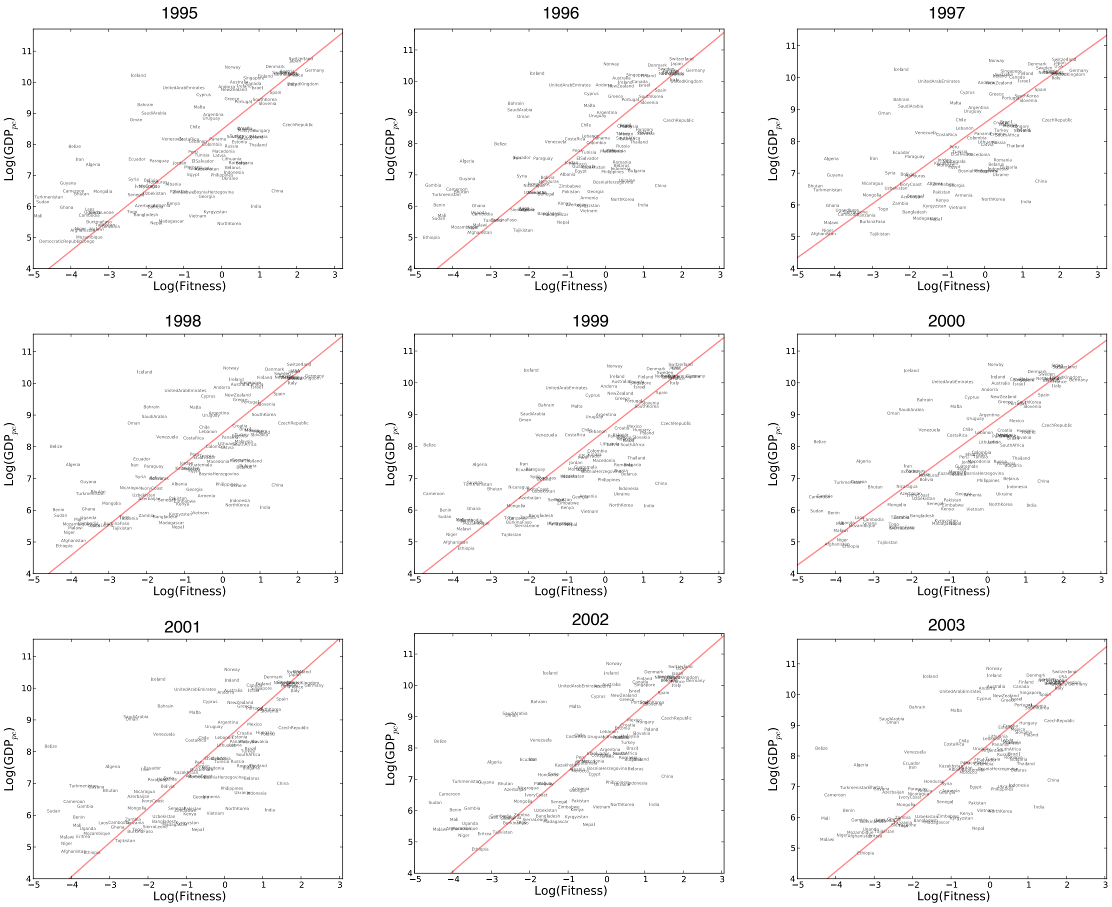

Figure S 1: **Countries in the fitness-income plane from 1995-2010**

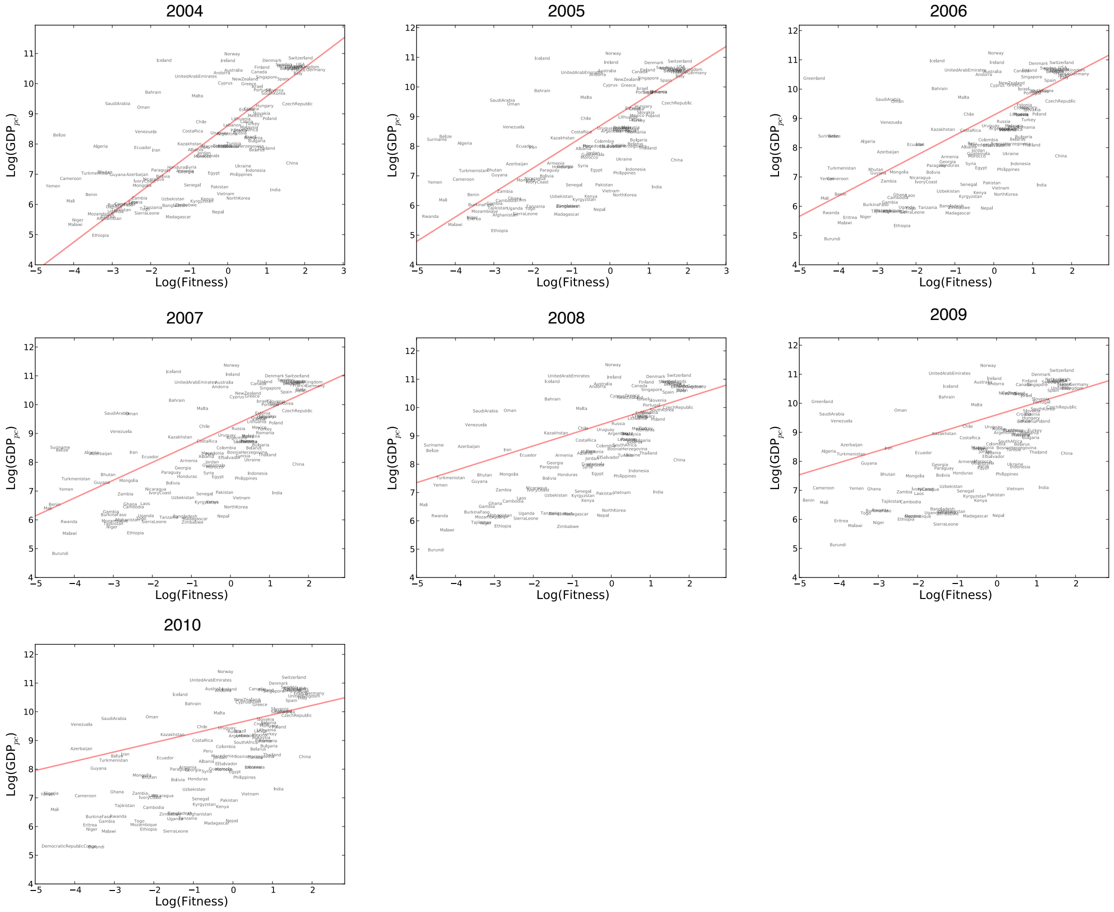

Figure S 2: **Countries in the fitness-income plane from 1995-2010**

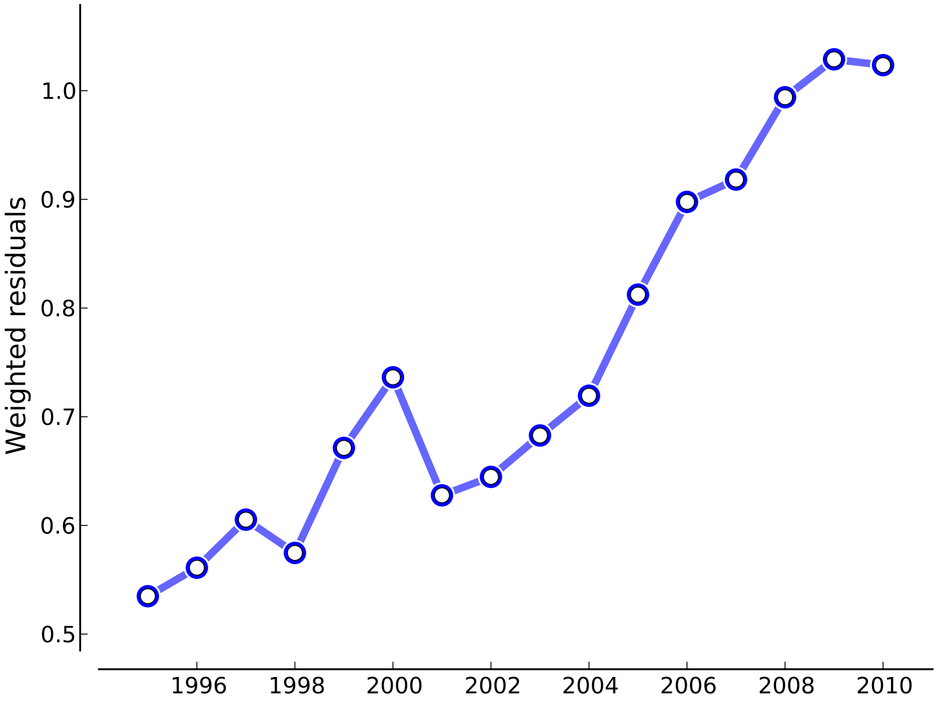

Figure S 3: **Time evolution of the residuals from the average line from 1995 to 2010**: we do not observe, over the period considered, a tendency towards an equilibrium situation in the fitness-income plane, i.e. a straight line. On the contrary, we observe a general increase of the residuals.

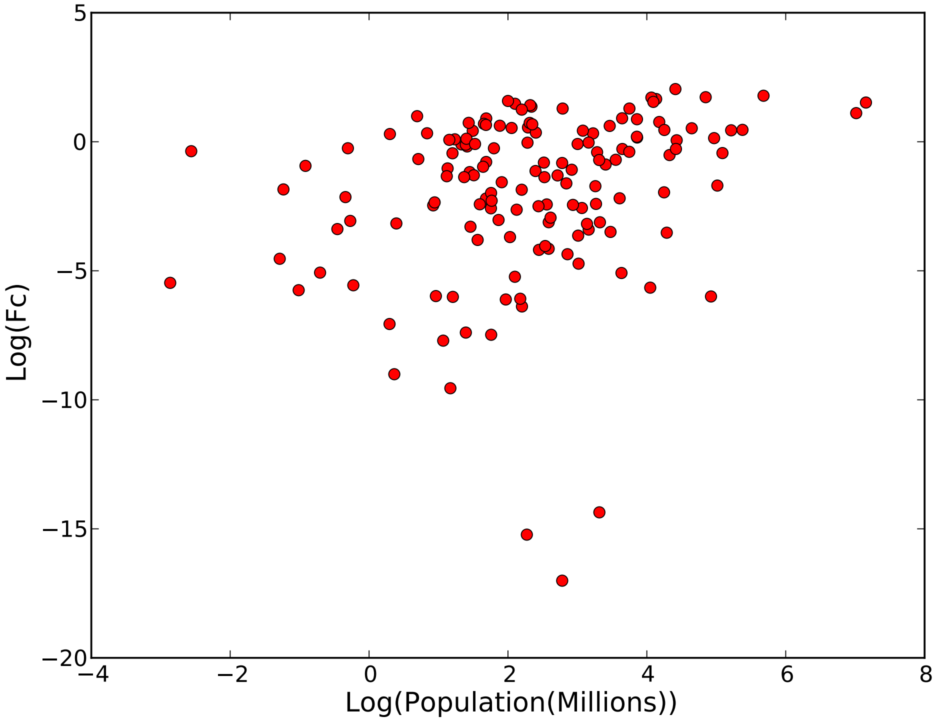

Figure S 4: **Log(Population) vs Log(Fitness) - 2004**. For the regression in which Log(Fitness) is the dependent variable and the Log(Population) the independent one, we obtain $R^2 = 0.06$. The sign of the coefficient estimated by the regression indicates a positive relationship of the residual dependence between these two variables.

On one hand, we observe that the proposed algorithm removes almost all trivial correlations among the fitness and the size/population of countries allowing for direct comparison of countries and supporting the observation that the monetary counterpart of the fitness is indeed the GDP *per capita* since the fitness almost uncorrelated with the number of inhabitants of a country. For comparison, we find that the population of countries accounts for $11 - 15\%$ of the variance of the diversification (the zero order iteration of the fitness) in the same range of years.

On the other hand, we can give an economic interpretation in terms of capabilities of the small residual fraction of the fitness' variance explained by the population of countries. We argue that population is a kind of capability and, even once the size effect of a country is removed by our method, it emerges that a residual part of the competitiveness of a country, as measured by the fitness, can be explained in terms of its population. The inspection of the sign of the correlation between the two variables indicates, coherently with the interpretation in terms of capabilities, a positive dependence of the fitness on the population. This results is also supported by the general belief that demographic aspects are one of the key necessary factors for the growth.

3 Standard regressive approach and heterogeneity.

We now discuss the results which would be obtained from a standard regressive approach in which we test a series of variables and their combination as regressors for the growth of GDP and of the GDP *per capita* over a period of 5, 10 and 15 years.

The regressions performed will be in the following form:

$$\log(GDP_{t+\Delta}) - \log(GDP_t) = c + \sum_i b_i \, x^{(i)} \tag{1}$$

where $\log(GDP_{t+\Delta}) - \log(GDP_t)$ is the log-return of the GDP, $\Delta = 5, 10, 15$ years, c and $\{b_i\}$ are the coefficients estimated in the regressions and $\{x^{(i)}\}$ are the set of regressors used.
In Table S1 we report the description of the variables used as regressors in the following sections.

Table S 1: Description of variables used as regressors in Eq. 1.

Name	Description
GDP	GDP at time t expressed in Trillions of USD
GDP_{pc}	GDP per capita at time t expressed in 10^3 USD
$\log(F)$	Logarithm of the fitness of countries
d	Signed vertical distance of the countries from the average line shown in Figs. S1 and S2 at time t. Countries above the line get a negative distance, countries below a positive one.

We report in the following the results of the regressions. Each line of the tables represents a different regression, the first column indicates the dependent variable of the regression, i.e. the log-return of the GDP, in the last column we report the percentage of variance of the log-returns explained by the regressors used and in the remaining columns we report the coefficients associated to the regressors. A missing value in one of the columns associated to a regressor means that this specific variable is not used in the regression.

Following standard symbols about significancy of regression results, we adopt the following convention for the p-values of the coefficients estimated by the regression: $^* = p-value < 0.05$, $^{**} = p-value < 0.01$ and $^{***} = p-value < 0.001$.

3.1 5-years prediction

On such time horizon we have three non-overlapping periods, namely 1995-2000, 2000-2005 and 2005-2010 to perform the regression. This corresponds to 439 observations considering the three periods all together.

Table S 2: Regressions for 5-years GDP growth forecast.

5 years	c	$\log(F)$	d	GDP(Trillions)	R^2
Return GDP	0.180	-0.0744^{**}			0.023
Return GDP	0.328^{**}	-0.0493^{*}		-0.399^{***}	0.069
Return GDP	0.440^{***}			-0.441^{***}	0.059
Return GDP	0.337^{***}		0.00241		0.000
Return GDP	0.453^{***}		0.0136	-0.443^{***}	0.059
Return GDP	0.166	-0.212^{***}	0.234^{***}	-0.312^{***}	0.108

Table S 3: Regressions for 5-years GDP per capita growth forecast.

5 years	c	$\log(F)$	d	GDP_{pc}	R^2
Return GDP_{pc}	0.178^{*}	-0.0423^{*}			0.009
Return GDP_{pc}	0.663^{***}	0.0116		-0.0487^{***}	0.126
Return GDP_{pc}	0.630^{***}			-0.0475^{***}	0.126
Return GDP_{pc}	0.296^{***}		0.0348		0.004
Return GDP_{pc}	0.639^{***}		0.0135	-0.0471^{***}	0.126
Return GDP_{pc}	0.654^{***}	0.00717	0.00557	-0.0481^{***}	0.127

3.2 10-years prediction

In that case, given the range of years investigated, we cannot find more than one period non-overlapping. Therefore we perform two separate regressions on the periods 1995-2005 and 2000-2010 composed of 146 observations each.

Table S 4: Regressions for 10-years GDP growth forecast.

$95-05$	c	$\log(F)$	d	GDP(Trillions)	R^2
Return GDP	0.411^{**}	-0.0935^{**}			0.055
Return GDP	0.608^{***}	-0.0627^{*}		-0.626^{***}	0.154
Return GDP	0.767^{***}			-0.700^{***}	0.131
Return GDP	0.557^{***}		-0.0546		0.013
Return GDP	0.716^{***}		-0.0376	-0.684^{***}	0.137
Return GDP	0.521^{***}	-0.175^{*}	0.144	-0.555^{***}	0.172

Table S 5: Regressions for 10-years GDP per capita growth forecast.

$95-05$	c	$\log(F)$	d	GDP_{pc}	R^2
Return GDP_{pc}	0.368^{***}	-0.0456^{*}			0.026
Return GDP_{pc}	0.591^{***}	-0.0209		-0.0257^{**}	0.070
Return GDP_{pc}	0.659^{***}			-0.0288^{**}	0.066
Return GDP_{pc}	0.460^{***}		-0.0106		0.000
Return GDP_{pc}	0.645^{***}		-0.0110	-0.0289^{**}	0.067
Return GDP_{pc}	0.329	-0.166	0.165	-0.00399	0.087

Table S 6: Regressions for 10-years GDP growth forecast.

$00-10$	c	$\log(F)$	d	GDP(Trillions)	R^2
Return GDP	0.532^{*}	-0.167^{**}			0.052
Return GDP	0.612^{*}	-0.154^{*}		-0.242	0.059
Return GDP	0.960^{***}			-0.372	0.017
Return GDP	0.855^{***}		-0.0358		0.001
Return GDP	0.939^{***}		-0.0286	-0.367	0.018
Return GDP	0.232	-0.487^{***}	0.495^{***}	-0.0507	0.135

Table S 7: Regressions for 10-years GDP per capita growth forecast.

$00-10$	c	$\log(F)$	d	GDP_{pc}	R^2
Return GDP_{pc}	0.513^{*}	-0.115^{*}			0.028
Return GDP_{pc}	1.558^{***}	-0.00291		-0.124^{***}	0.227
Return GDP_{pc}	1.566^{***}			-0.125^{***}	0.227
Return GDP_{pc}	0.786^{***}		0.0444		0.002
Return GDP_{pc}	1.56^{***}		-0.0119	-0.125^{***}	0.227
Return GDP_{pc}	1.73^{***}	0.0690	-0.0926	-0.137^{***}	0.227

3.3 15-years prediction

For this time horizon, we have only one possible set of data (1995-2010) corresponding to 146 observations.

Table S 8: Regressions for 15-years GDP growth forecast.

15 years	c	$\log(F)$	d	GDP(Trillions)	R^2
Return GDP	0.579^{*}	-0.182^{**}			0.069
Return GDP	0.665^{*}	-0.168^{**}		-0.275^{***}	0.075
Return GDP	1.09^{***}			-0.471	0.019
Return GDP	0.889^{***}		-0.0850		0.010
Return GDP	0.992^{***}		-0.0740	-0.440	0.027
Return GDP	0.349	-0.575^{***}	0.526^{***}	-0.0165	0.152

Table S 9: Regressions for 15-years GDP per capita growth forecast.

15 years	c	$\log(F)$	d	GDP_{pc}	R^2
Return GDP_{pc}	0.496^{*}	-0.128^{*}			0.038
Return GDP_{pc}	1.461^{***}	-0.0214		-0.112^{***}	0.202
Return GDP_{pc}	1.531^{***}			-0.114^{***}	0.201
Return GDP_{pc}	0.771^{***}		-0.0172		0.000
Return GDP_{pc}	1.50^{***}		-0.0188	-0.115^{***}	0.202
Return GDP_{pc}	1.37^{**}	-0.0718	0.0575	-0.104^{**}	0.203

The conclusions which can be derived from these results are twofold. On one hand, all regressions performed appear to have a very poor predictive power on country growth. On the other hand, the coefficients found are not consistent across different regressions. In the 10-years growth case, the predictive power of the regression is dramatically dependent on the time interval considered and the signs of the coefficients estimated show inconsistencies (see also [3] for sign inconsistencies in the use of regressions for forecasting GDP growth). As discussed in the main text, a regression-based approach answers to the question of unveiling a general homogeneous behavior of the system. Making a parallel with weather forecast, regressions, in such context, would correspond to ask how the weather is in the world tomorrow. Clearly this question is ill-defined, the correct question for the atmosphere dynamics is how weather will be in a specific region/city. In a similar way, we argue that the correct question in the assessment of the growth forecast is the expected growth of a country in a specific economic regime. The expected evolution of countries is dependent on the economic regime in which the country is found to be, as the atmospheric dynamics is dependent on the region we are considering. In this sense, we are in a scenario – the heterogeneous dynamics of economic complexity – where we face issues similar to those encountered in dynamical systems.

3.4 Robustness of the heterogeneous regime in the Fitness-Income plane

A necessary condition to obtain a meaningful and successful forecast scheme is that the heterogeneity of the dynamics must show a stability in time. As confirmed in Figs. S5-S6, the two regions of the economic dynamics – laminar and chaotic – result to be robust in time. In both figures, we report in red the coarse-grained dynamics discussed in the main text and obtained using the full dataset from 1995 to 2010 for comparison. As a future extension of the present work on a longer dataset, we plan to investigate the evolution of the boundary between the laminar and the chaotic regime.
It is also interesting to observe the small discrepancies among the economic dynamics in the three time windows considered in Fig. S5. This figure supports the idea that the *proper* scale to evaluate the EDs is in the range $10-15$ years in order to average over short time effects due to specific moments of economic cycles. In this sense, the *selective predictability scheme* has a natural time horizon for country evolution forecasting around 10 years, as also confirmed in the following sections.

We also tested the robustness of all analyses with respect to shifts of the grid and different size of the coarse graining and found a substantial independence of our findings on the details of the coarse graining procedure.

3.5 Measure of concentrations

A natural candidate to measure the dispersion of the EDs would be the entropy, which can be indeed seen as a measure of the concentration of the information of a distribution. However, the correct estimation of the entropy of the EDs critically relies on a robust estimation of the empirical frequencies of the EDs. Simple numerical simulations on toy models reveal that, given the small *typical* level of statistics of the present analysis, the measure of the entropy of the ED would strongly depends on the finite size effects, which affects the empirical frequencies.

We therefore define an *average* measure of concentration, which does not rely on the estimation of the empirical frequencies of the ED as it follows

$$\mathcal{C} = \frac{n^{(i)}_{boxes}/N^{(i)} - 1/N^{(i)}}{1 - 1/N^{(i)}} \tag{2}$$

where $N^{(i)}$ and $n^{(i)}_{boxes}$ are respectively the number of events giving rise to the $i-th$ ED and the number of boxes in which these $N^{(i)}$ evolved after a given time lag. The $\mathcal{C}$ is a normalized concentration measure since it can range from 0 to 1. In addition, the present measure has the advantage to estimate the concentration of the EDs independently on the features of the ED. Instead entropy mixes these two aspects. As a second step of our analysis, to measure how broad or peaked the distributions arising from the boxes are, we use a standard measure of concentration in economics, the normalized Herfindahl index H^*:

$$H^* = \frac{H - 1/N}{1 - 1/N} \tag{3}$$

which ranges from 0 to 1 and Herfindahl index H is defined as:

$$H = \sum_i p_i^2, \qquad \sum_i p_i = 1 \tag{4}$$

As shown in Fig. S7, although the EDs of boxes from the laminar regime have very similar level of concentration as measured by $\mathcal{C}$, the Herfindahl index of the boxes from the laminar regime shows, in its turn, a non-trivial degree of heterogeneity of the features of the EDs. We recall once again that, independently on the value of the Herfindahl index, these EDs are characterized by an evolution in which the number of final occupied boxes is very small compared to the number of events.

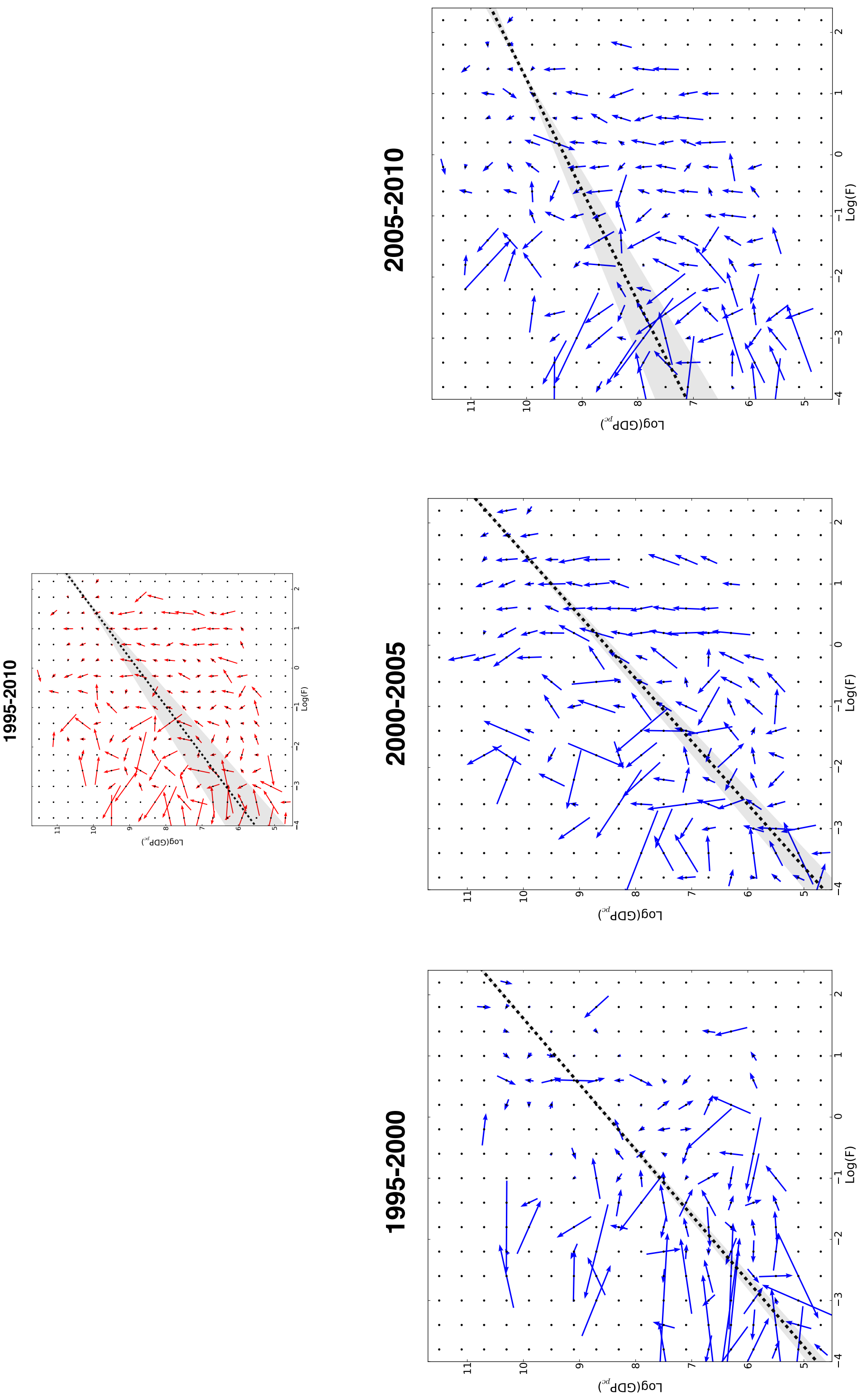

Figure S 5: **Coarse grained dynamics in the Fitness-Income Plane performed on time windows of 5 years**. Top panel: for comparison, coarse grained dynamics from 1995 to 2010.

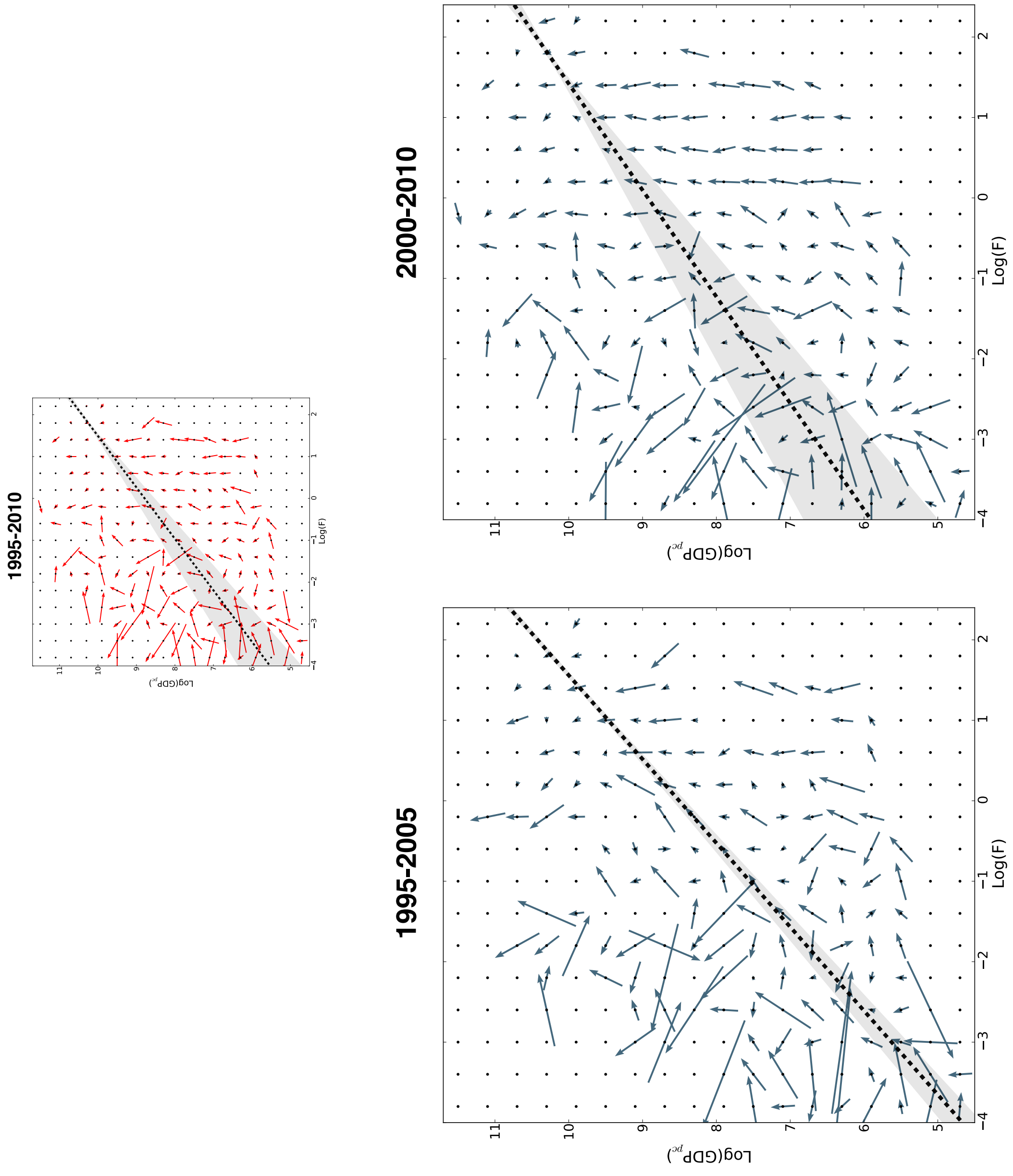

Figure S 6: **Coarse grained representation of the dynamics in the Fitness-Income Plane performed on time windows of 10 years**. Top panel: for comparison, coarse grained dynamics from 1995 to 2010.

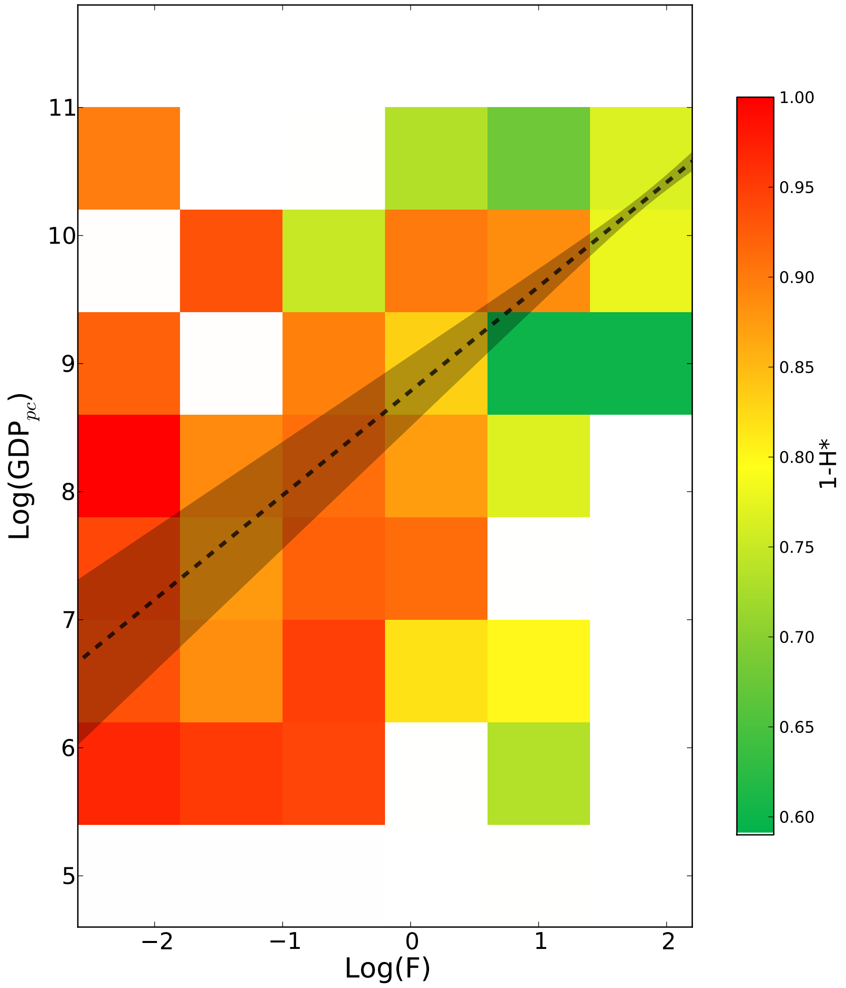

Figure S 7: **Normalized Herfindahl index of the EDs in the laminar regime, time lag**$= 10$ **years**. The dynamics of economic complexity of countries in the fitness-income plane also exhibits a non-trivial heterogeneity in the features of the EDs in the laminar regime. $H^* = 1$ corresponds to the case in which all the events are concentrated in a single box while $H^* = 0$ when the distribution is uniform. We plot $1 - H^*$ in order to obtain a quantity which, as the entropy and $\mathcal{C}$, is 0 when all events are in a single box. We report the measure for boxes with at least 4 events.

3.6 Estimation of the EDs

As illustrated in Fig. S8, each ED is obtained by considering all countries originating from a box and recording their positions after a certain time lag (in our case 5 and 10 years). For a finer forecast resolution, the grid in which the positions of the evolved countries are recorded has a smaller box size. In our case, we consider a grid for evolved distributions whose box size is the half of the grid defining the starting box of our scheme.

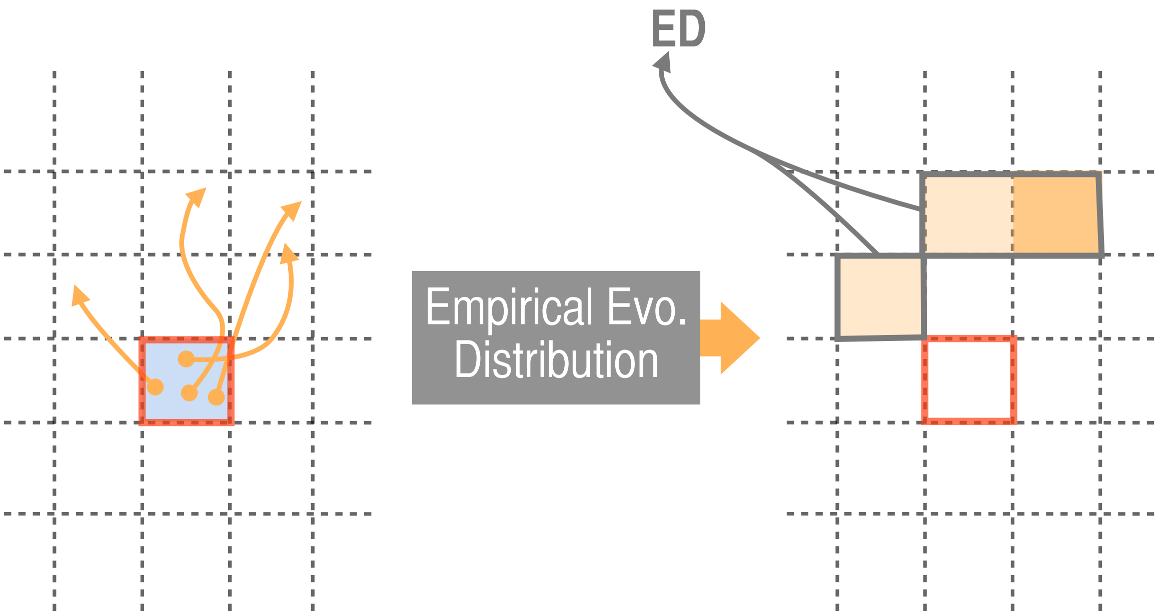

Figure S 8: **Illustration of how the EDs are estimated** .

3.7 *Selective predictability scheme*: 5-years prediction

We report in this section the *selective predictability scheme* in the case in which the EDs are built tracking the 5-years evolution in the fitness-income plane. In Fig. S9, we show a selection of EDs, the red squares and dots indicate the starting box, while in Fig. S10 we show the concentration as measured by $\mathcal{C}$ (left panel) of the EDs for all boxes. In the right panel, we also report the entropy for each ED since we have a larger statistics and the entropy estimation is less biased by the finite size effects occurring in the reconstructions of the empirical frequencies. We observe that entropy behavior confirms all the conclusions based on the analysis of $\mathcal{C}$ and Herfindahl index. As in the 10-years case, we show in Fig. S11 the normalized Herfindahl index for all EDs highlighting the heterogeneity of these distributions even in the laminar regime.

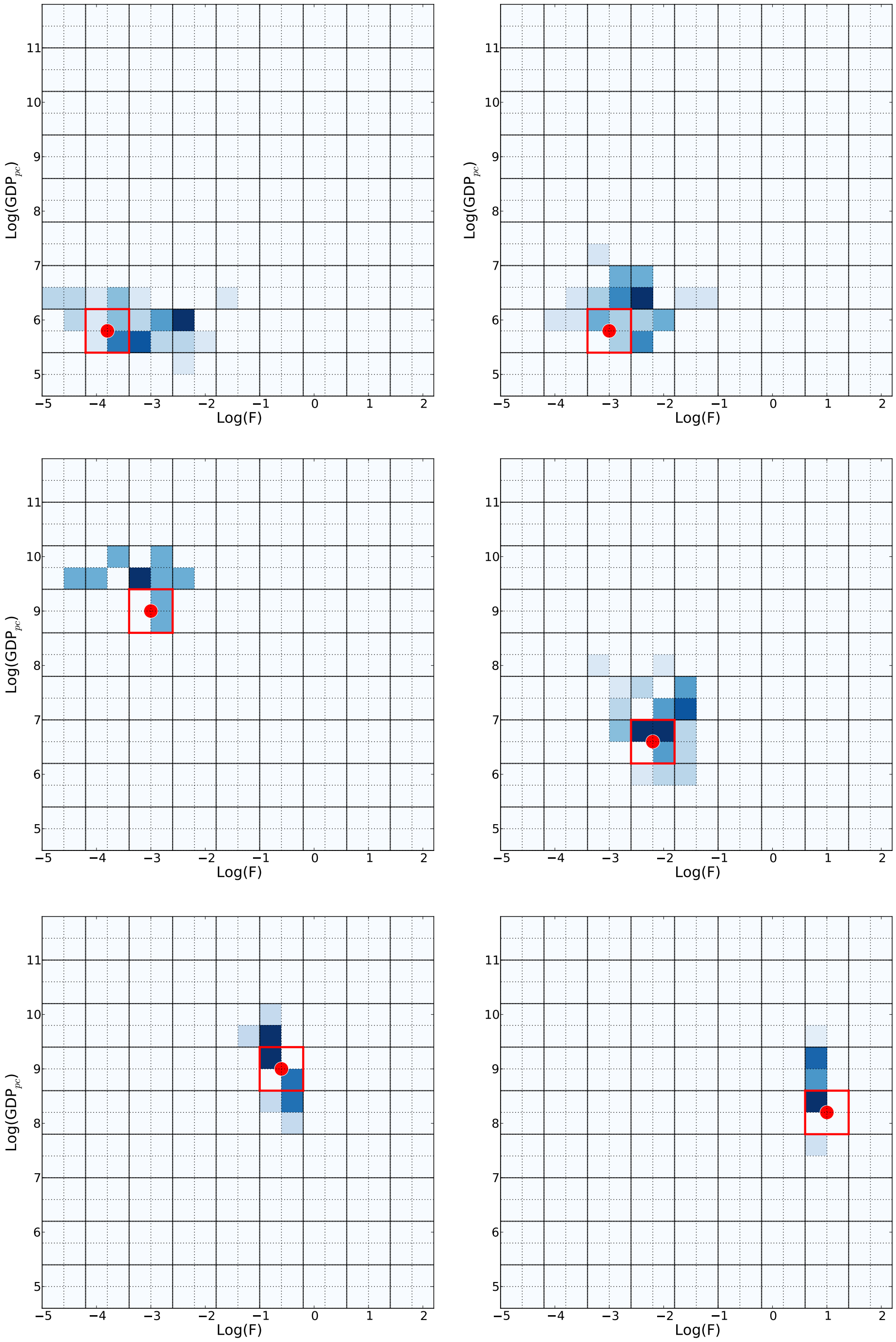

Figure S 9: **Selection of EDs from both the chaotic and laminar regime**. The time lag is equal to 5 years.

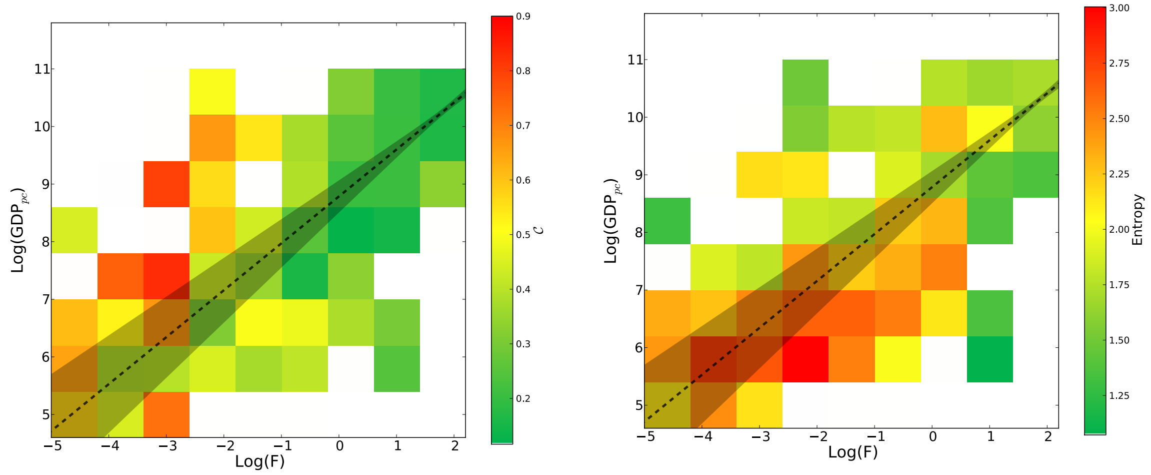

Figure S 10: **Measures of concentration for EDs**. The time lag is equal to 5 years. (Left panel) The 5-years case of the *selective predictability scheme* appears to be similar to the 10-years case: we can divide the dynamics in two regions, laminar characterized by EDs poorly dispersed and chaotic where EDs are very broad. (Right panel) In that case we have enough statistics to show significant estimation of the entropy which confirms the existence of two regions with very different features of the EDs. The entropy analysis also confirms the heterogeneity inside the laminar regime where, even if poorly dispersed, two kinds of EDs are observed: very peaked on only one box (high value of normalized Herfindahl index) and more uniformly distributed (low value of normalized Herfindahl index), see also Fig. S11. We report the concentration measure for boxes with al least 5 events.

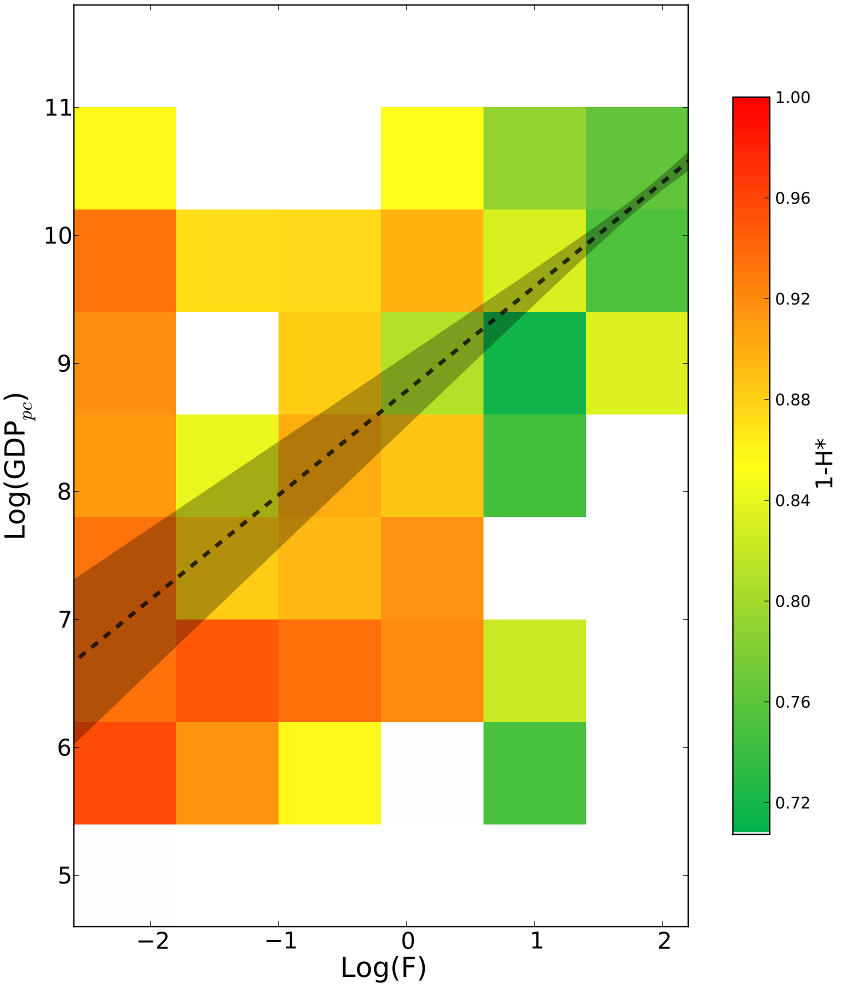

Figure S 11: **Normalized Herfindahl index of the EDs in the laminar regime, time lag= 5 years**. The dynamics of economic complexity of countries in the fitness-income plane also exhibits a non-trivial heterogeneity in the features of the EDs in the laminar regime. $H^* = 1$ corresponds to the case in which all the events is concentrate in a single box while $H^* = 0$ when the distribution is uniform. We plot $1 - H^*$ in order to obtain a quantity which, as the entropy and $\mathcal{C}$, is 0 when all events are in a single box. We report the index for boxes with at least 5 events.

3.8 *Selective predictability scheme*: 10-years prediction

We report in this section the *selective predictability scheme* in the case in which the EDs are built tracking the 10-years evolution in the fitness-income plane. In Fig. S12 we show a selection of EDs. As previously discussed, we do not have a reliable estimation of the entropy of the EDs due to the small statistics we are considering, however, for the sake of completeness we report the entropy for each box in Fig. S13 .

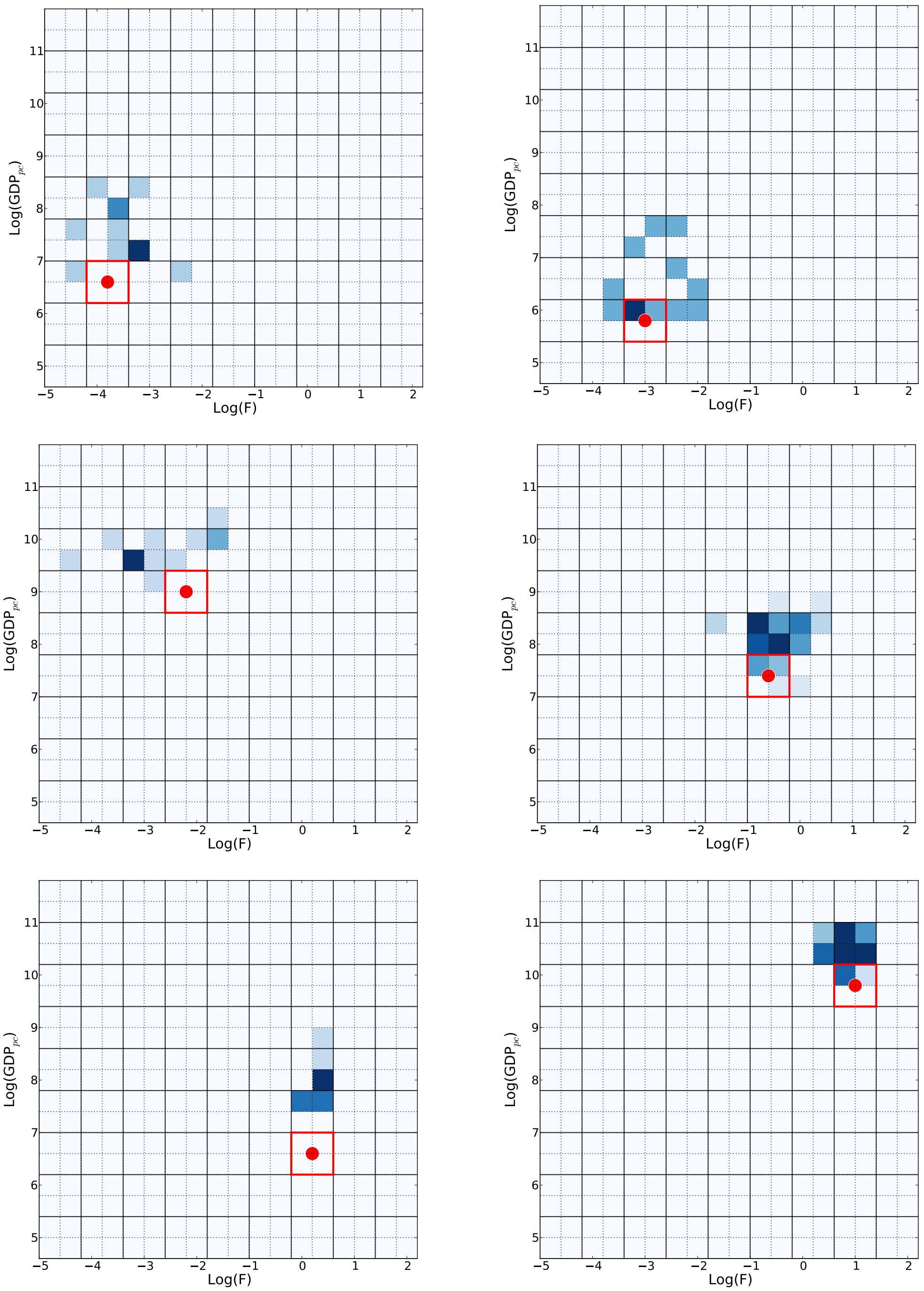

Figure S 12: **Selection of EDs from both the chaotic and laminar regime**. The time lag is equal to 10 years.

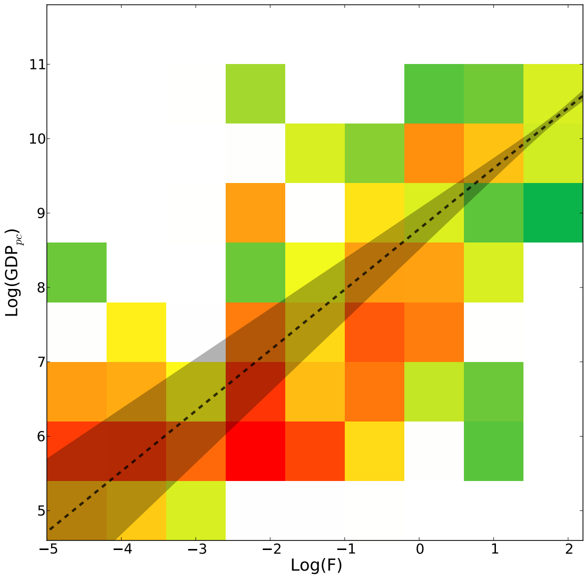

Figure S 13: **Entropy of the EDs for 10-years *selective predictability scheme*.** Differently from the 5-years case, entropy estimation may be strongly biased by the small statistics we are considering.

4 Backtesting ED-based forecasting scheme

The stability in time of the coarse grained dynamics and the robust patterns observed measuring the concentration of EDs ground the existence of two kinds of regime for the dynamics of the economic complexity and, consequently, the *selective predictability scheme*. As a final analysis to support our forecasting scheme for economic growth, we perform a backtesting of our method – backtesting represents a standard way to test a forecasting scheme, see for instance [4] for financial application.

Given the limited time window under investigation, we can only perform a backtest of the 5-years *selective predictability scheme*. We estimate the 5-years EDs using the evolution of countries from 1995-2000 and then we test the rate of success of the prediction of the position of countries in 2010 given their position in 2005 according to the EDs obtained in training time period.

In Fig. S14 we report the rate of success measured as the ratio of predicted events and the total cases. In the left panel, we report the case in which we consider boxes with at least 2 events, while in the left with at least 3. Despite the small statistics of the test, it appears that the ED from the laminar regime has a significant and systematically higher rate of success. We stress once again that, even if the ratio of success of two EDs, one from the laminar regime and one from the chaotic regime, were the same, the forecast of the evolution of the country in the first case would correspond to indicate a much smaller area in the fitness-income plane in which we expect to observe the country.

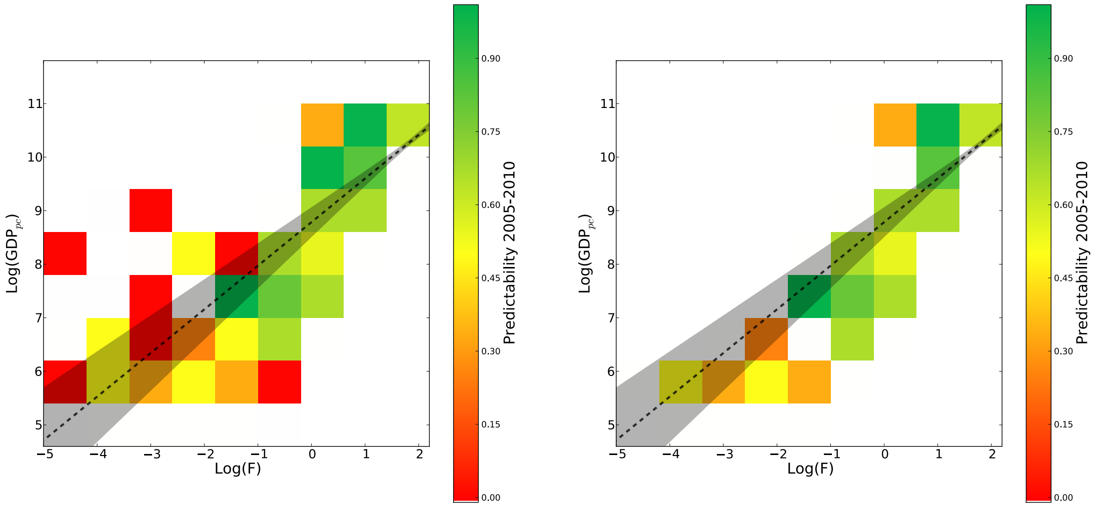

Figure S 14: **Backtest of the 5-years *selective predictability scheme***. (Left panel) We consider boxes with at least 2 events, (right panel) events with at least 3 events. In both cases the conclusion that the laminar regime exhibits a much higher degree of predictability is confirmed.

4.1 2010's fitness and GDP *per capita* forecast

Given the ED estimated from the training set from 1995 to 2005 and given 2005's fitness and GDP *per capita*, the forecast of 2010's evolution is illustrated in Fig. S15. We calculate the center of mass of starting points for each ED (B_1 in Fig. S15) and the center of mass of the evolved points (B_2 in Fig. S15). For each ED, we then compute the vector associated to the displacement of the center of mass as shown in panel a) of Fig. S15. For each country, we apply to the 2005's position in the fitness-income plane the displacement vector previously calculated depending on the box in which the country is (Fig. S15 (panel b)) . The relative error reported in Fig. 4 of the main paper is simply the difference between this forecast and the realized 2010's GDP *per capita* normalized with the realized GDP *per capita*. As discussed in the main paper, we believe that the systematic under estimation is due to a training set shorter than the length of an economic cycle.

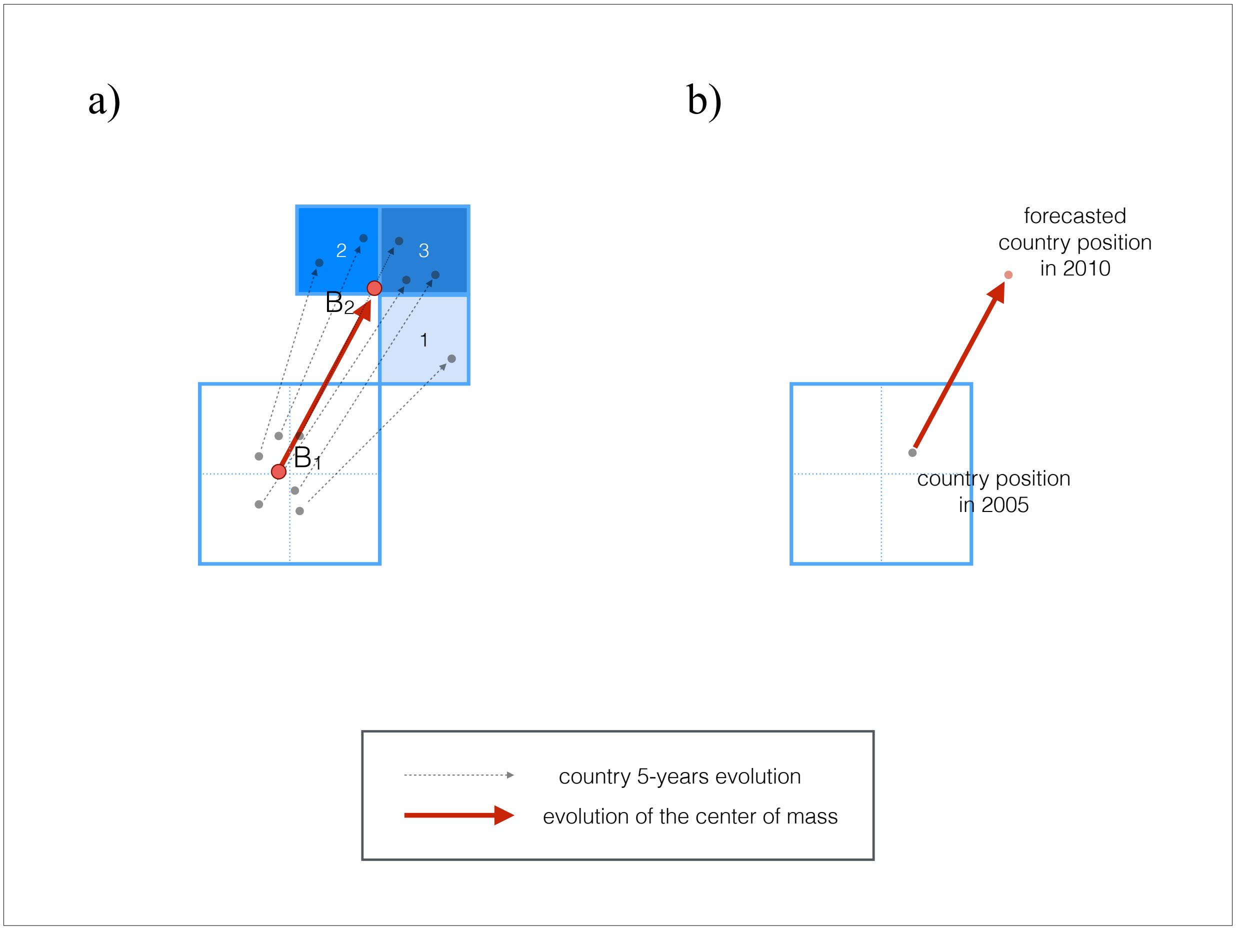

Figure S 15: **Illustration of the forecast method for 2010**.

References

[1] Tacchella, A., Cristelli, M., Caldarelli, G., Gabrielli, A. & Pietronero, L. A new metrics for countries' fitness and products' complexity. *Scientific Reports on Nature* **2**, 723 (2012). URL `http://www.nature.com/srep/2012/121010/srep00723/full/srep00723.html`.

[2] Cristelli, M., Tacchella, A., Gabrielli, A., Caldarelli, G. & Pietronero, L. Measuring the intangibles: a metrics for the economic complexity of countries and products. *PLOS ONE* **8(8):e70726** (2013). URL `http://www.plosone.org/article/info%3Adoi%2F10.1371%2Fjournal.pone.0070726`.

[3] Hidalgo, C. & Hausmann, R. The building blocks of economic complexity. *Proceedings of the National Academy of Sciences* **106**, 10570–10575 (2009).

[4] Park, C.-H. & Irwin, S. H. What do we know about profitability of technical analysis. *Journal of Economic Surveys* **21**, 786826 (2007).
